# Fungal Diversity, Toxigenic Potential, and Multi-Mycotoxin Occurrence in Fresh and Dried Chili Peppers from Retail Markets in Guangzhou, China

**DOI:** 10.3390/toxins18040154

**Published:** 2026-03-24

**Authors:** Maryam Tavakol Noorabadi, Ishara S. Manawasinghe, Jiayu Xu, Caiqing Zhao, Naghmeh Afshari, Wei Dong, Antonio Francesco Logrieco, Kevin D. Hyde

**Affiliations:** 1 Innovative Institute for Plant Health/Key Laboratory of Green Prevention and Control on Fruits and Vegetables in South China, Ministry of Agriculture and Rural Affairs, Zhongkai University of Agriculture and Engineering, Guangzhou 510225, China; maryam.tavakol65@yahoo.com (M.T.N.); zhaocaiqing2022@163.com (C.Z.); dongwei0312@hotmail.com (W.D.); 2 Institute of Plant Protection, Beijing Academy of Agriculture and Forestry Sciences, Beijing 100097, China; ishara9017@gmail.com; 3 Xianghu Laboratory, Biomanufacturing Institute, Hangzhou 311231, China; xujiayu@xhlab.ac.cn; 4 Department of Biology, Faculty of Sciences, Chiang Mai University, Chiang Mai 50200, Thailand; naghmeh.afshar20@gmail.com; 5 Center of Excellence in Fungal Research, Mae Fah Luang University, Chiang Rxai 57100, Thailand; 6 Key Laboratory of Phytochemistry and Natural Medicines, Kunming Institute of Botany, Chinese Academy of Sciences, Kunming 650201, China

**Keywords:** fungal contamination, *Capsicum annuum*, *Fusarium*, *Penicillium*, mycotoxins, LC–MS/MS

## Abstract

This study provides a combined profile of fungal isolates from fresh and dried chili peppers in markets in Guangzhou. Multilocus sequence analysis revealed a wide variety of species, seven of which were reported for the first time from chili pepper (*F. annulatum, F. compactum, F. pernambucanum, F. ramsdenii,* and *F. tardichlamydosporum*, *P. citrinum* and *P. steckii*). In this research work, quantitative determination using targeted LC–MS/MS of dried chili peppers showed a significantly higher frequency of contamination and higher toxin concentrations than fresh samples. The predominant mycotoxins in dried peppers were DON and FB_1_, which were present in all the samples at mean levels of 0.56 µg/g and 0.067 µg/g, respectively. AFB_1_ and OTA were present in all dried samples but were detected only occasionally in fresh peppers. ZEN and CIT were detected at lower concentrations, but more prevalent among dried products (63.6% and 81.8% of all samples, respectively). The aflatoxin B_1_ (AFB_1_) level of 180 µg/kg in dried chili samples was 36 times above the EU maximum limit (5 µg/kg), and the OTA level reached 54 µg/kg, exceeding the EU limit by a factor of 2.7 (20 µg/kg). Statistical analysis also showed that all six mycotoxins were statistically higher in dried pepper than in fresh pepper. In vitro evaluation demonstrated that certain *Fusarium* isolates synthesized FB_1_. At the same time, *Penicillium* species, including *P. citrinum* and *P. steckii*, consistently produced citrinin, confirming the strong influence of growth substrate on toxin biosynthesis. The frequent occurrence and elevated levels of regulated mycotoxins highlight significant public health concerns and underscore the need for improved postharvest handling and drying practices. These findings provide critical baseline data linking fungal diversity with toxin production dynamics, developing essential guidance for targeted mitigation strategies.

## 1. Introduction

Dried and fresh chili peppers (*Capsicum annuum* L.) hold significant prominence within the culinary, agricultural, and medicinal sectors in China, where they contribute not only to dietary diversity but also to national economic development. Their production and utilization enhance local cuisine while supporting extensive agricultural activities and food-processing industries [1,2]. As the world’s largest producer of fresh chili peppers, China has established a robust agricultural infrastructure for cultivating this crop, achieving an annual yield of over 18 million tons [2]. Moreover, chili pepper cultivation plays a vital role in rural revitalization initiatives, providing farmers with profitable opportunities and strengthening regional economies [1]. The distinction between dried and fresh chili peppers further reflects their cultural and culinary significance across China. Fresh peppers are integral to traditional dishes such as hot pots, where their characteristic flavor profiles and varying pungency levels contribute to gastronomic richness [3]. Their spiciness results primarily from capsaicinoids, especially capsaicin and dihydrocapsaicin, which impart both pungency and notable medicinal properties [4,5].

The cultivation of chili peppers across China mirrors the country’s diverse agro-ecological landscape and showcases the remarkable adaptability of this crop under evolving climatic conditions [2]. Distinct climatic zones enable the production of region-specific pepper varieties, such as those from Sichuan and Yunnan, which are deeply embedded in local cuisines and cultural identity [6]. These geographical and climatic differences shape plant physiology, flavor development, and resilience to environmental stressors, while also influencing disease prevalence and pathogen behavior. Environmental factors, including elevated humidity, high temperatures, and substantial rainfall, play crucial roles in determining the incidence of fungal pathogens and the accumulation of associated mycotoxins [7,8]. Chili peppers grown in high-humidity regions are particularly susceptible to fungal invasion, highlighting the influence of climate-driven ecological pressures on contamination risk [9].

Within this broader agricultural context, chili pepper is recognized as a commercially important crop that is highly vulnerable to fungal colonization and subsequent mycotoxin production. Fungal infections can occur throughout the entire production chain—from field growth and harvest to drying, processing, and storage—making chili peppers susceptible at multiple critical points [10,11]. Improper postharvest handling practices, such as insufficient drying or extended storage under humid conditions, further exacerbate the risk of fungal proliferation and toxin accumulation [12]. Processed chili products, such as crushed or powdered red pepper, are even more susceptible to contamination than whole fruits, particularly under suboptimal storage conditions with elevated moisture levels [13,14,15].

Mycotoxins, harmful secondary metabolites produced primarily by filamentous fungi, represent a major global food-safety challenge due to their presence in a wide range of agricultural commodities. These toxins pose severe risks to human and animal health and contribute to significant economic losses worldwide [16,17,18,19,20,21]. The principal toxigenic genera associated with food contamination include *Aspergillus*, *Fusarium*, and *Penicillium*, alongside lesser contributors such as *Alternaria* and *Cladosporium*, all of which synthesize diverse toxins under favorable environmental conditions [22,23,24,25]. Although more than 300 mycotoxins have been identified, only a subset, including aflatoxins, trichothecenes, zearalenone, fumonisins, ochratoxins, and patulin, are considered highly hazardous due to their potent toxicity and frequent occurrence in food systems [18,26]. The health risks posed by these toxins are severe and well-documented. Aflatoxin B_1_ (AFB_1_) is a potent hepatocarcinogen, classified by the International Agency for Research on Cancer as a Group 1 human carcinogen, with its frequent occurrence in spices representing a major global food safety burden [27]. Ochratoxin A (OTA), commonly detected in dried commodities, is a nephrotoxin and a possible human carcinogen (IARC Group 2B), with chronic exposure linked to kidney disease in humans [28]. Furthermore, co-exposure to AFB_1_ and fumonisin B_1_ (FB_1_) has been shown to induce synergistic hepatotoxic and carcinogenic effects in animal models, suggesting that co-contamination may pose a significantly greater risk to human health than exposure to either toxin alone [29,30]. These contaminants readily enter the food chain either directly through mold-infected crops or indirectly via animal-derived products containing toxin residues or metabolites [31,32,33]. Major foodborne mycotoxin classes include trichothecenes—such as deoxynivalenol (DON) and T-2 toxins; aflatoxins (AFB_1_, AFB_2_, AFG_1_, and AFG_2_); ochratoxins (OTA and OTB); zearalenone (ZEN); fumonisins (FB_1_, FB_2_, and FB_3_); patulin (PAT); and citrinin (CIT). More recently, emerging mycotoxins, including Alternaria toxins (AOH, AME, TeA, TEN, and ALT), enniatins (ENNA, ENNA1, ENNB, and ENNB1), and beauvericin (BEA), have gained attention due to their rising prevalence and uncertain toxicological profiles [34].

A significant challenge In modern mycotoxin research is the widespread co-occurrence of multiple toxins within the same commodity. Multi-mycotoxin contamination is now recognized as a critical food-safety issue because such toxins frequently coexist, may display additive or synergistic effects, and often withstand degradation during conventional food processing [35]. This is a particularly high risk in spices such as chilies. Market monitoring data indicates a high susceptibility potential, as *Capsicum*-containing products are frequently among the most contaminated species worldwide, especially in hot climates [36]. Critically, contamination is often multifaceted. A significant number of co-contaminated products are also reported in studies specifically conducted on chili-based or chili-containing products. For example, studies on fresh sweet peppers and resulting products showed that many samples were contaminated with several toxigenic fungi (*Aspergillus*, *Fusarium*, and *Alternaria*) and their associated mycotoxins, such as aflatoxins and ochratoxin A [37]. Such widespread co-occurrence makes routine exposure combinatorial and creates complexities in risk assessment.

Their co-occurrence has been documented in cereals, medicinal plants, chili-based products, and animal feed, leading to cumulative health risks for both humans and livestock [35,38]. Therefore, understanding interactions among co-occurring mycotoxins and accurately assessing their combined toxicity is essential for effective risk evaluation and public health protection [39,40,41]. Owing to suboptimal drying and storage, dried chilies and spices are high-risk commodities. A targeted monitoring study in Myanmar found that 56.1% of dried chili samples exceeded the EU limit for aflatoxins, and ochratoxin A was detected in 91% of the samples [42]. In Italian sweet pepper, there was multiplex mycotoxin contamination, with 100% of the samples containing between 2 and 16 mycotoxins, including OTA (51% occurrence) and aflatoxins (31% occurrence), highlighting serious problems in drying and processing [37]. Dried chili peppers are among the most vulnerable to multi-mycotoxin contamination. Market surveys based on scientific research demonstrate widespread co-occurrence of aflatoxins, ochratoxin A, and sterigmatocystin, with a large proportion of samples exceeding the EU maximum limits for aflatoxin B_1_ (5 µg/kg) and total aflatoxins (10 µg/kg), especially in processed products such as powder and flakes [43].

To address the limited understanding of how fungal diversity and mycotoxin contamination interact across chili pepper products, this study undertakes a comprehensive assessment of toxigenic fungi associated with fresh and dried chili peppers in Guangzhou. By integrating morphological characterization, multilocus molecular identification, and LC–MS/MS–based toxin profiling, the research aims to characterize species-specific toxigenic potential, elucidate patterns of co-contamination among multiple mycotoxins, and compare contamination risks between fresh and dried peppers. The study aims to assess substrate-dependent production of fumonisin B_1_ by selected *Fusarium* species on PDA and citrinin production by *Penicillium* on CYA and YES media, thereby providing further insight into the influence of growth conditions on toxin biosynthesis and contributing to a more nuanced understanding of potential food-safety risks. This study represents the first comprehensive integration of multilocus phylogeny and quantitative multi-mycotoxin assessment for retail chili peppers in southern China.

## 2. Material and Methods

### 2.1. Survey of Markets for Dried and Fresh Peppers with Pathogen Symptoms

From 2023 to 2025, a comprehensive market-based sampling survey was conducted across 10 major agricultural hubs in Guangzhou city (Table 1), to identify fungal infections in both fresh and dried chili peppers. Sampling was carried out directly in local markets, where fresh peppers were inspected for infection symptoms, including sunken lesions, chlorosis, fruit rot, and mycelial growth. In contrast, dried peppers were examined for mold growth, discoloration, and shriveling. Fresh peppers were collected during two primary harvesting seasons (April–October), and dried peppers were sampled year-round. Markets were selected based on their significance in regional chili distribution and historical reports of fungal infection. A total of 500 symptomatic samples (200 fresh and 300 dried) were collected from market vendors, with emphasis on high-humidity regions favorable for fungal proliferation. For the mycotoxin analysis, 66 samples (33 dried and 33 fresh) were analyzed in a single LC-MS/MS run to quantify all mycotoxins. Selection was purposeful: samples represented all markets, including fresh-dried pairs, and were of the same variety with visible fungal symptoms to optimize fresh-dried comparisons. All samples were collected aseptically to prevent cross-infection; fresh infected fruits were sealed in sterile polyethylene bags. Dried samples were placed in breathable paper envelopes to prevent moisture accumulation. Each composite sample (500 g) was labeled with its source, coordinates, collection date, and observed symptoms. Subsamples from multiple vendors within each market ensured representative coverage. Samples were transported in temperature-controlled containers at 4 °C to maintain quality, then categorized into symptomatic and asymptomatic groups, surface cleaned, and stored at –20 °C for subsequent fungal isolation and molecular analysis. Water activity (aw), moisture content, and storage time were not measured for samples, as this market-based survey prioritized fungal isolation and mycotoxin quantification over monitoring post-harvest parameters. Information on storage conditions before purchase was unavailable from vendors.

### 2.2. Fungi Isolation and Purification

Fresh pepper fruits exhibiting disease symptoms were surface-sterilized by immersion in 70% ethanol for 30–60 s, followed by 2–3 rinses in sterile distilled water. When necessary, samples were additionally treated with 0.5–1% sodium hypochlorite for 1–2 min to remove surface contaminants. Sterilized tissues were blotted dry under aseptic conditions, and small sections from lesion margins were placed onto potato dextrose agar (PDA) amended with chloramphenicol (50 mg/L) to suppress bacterial growth. Plates were incubated at 25–28 °C, and emerging fungal colonies were purified through successive hyphal tip transfers until uniform cultures were obtained. Dried pepper samples were first rehydrated in moisture chambers. Pepper fragments were placed on sterile supports above damp filter paper to maintain high humidity. After fungal structures emerged, the fragments were transferred onto PDA plates amended with chloramphenicol (50 mg/L). For purification, distinct colonies growing from the tissue were isolated by hyphal tip transfer to obtain pure cultures. Actively growing colonies were selected, and small agar blocks containing peripheral hyphal growth were excised under a stereomicroscope using a sterile scalpel or needle. The excised hyphal tips were transferred onto fresh potato dextrose agar (PDA) plates and incubated at 25–28 °C. This procedure was repeated two to three times to ensure the elimination of contaminating microorganisms. Purity of the isolates was confirmed by examining colony morphology and microscopic characteristics at each transfer stage. Verified pure cultures were maintained on PDA slants at 4 °C for short-term use and preserved in 15% glycerol at –80 °C for long-term storage. Only *Fusarium* and *Penicillium* were analyzed in detail, as they are the main mycotoxin producers in chili peppers, and other genera were recorded collectively.

### 2.3. Morphological Characterization

Representative isolates of each fungal species were cultured on PDA at 25 ± 1 °C for five days to promote active mycelial growth. From the advancing margins of these colonies, 7 mm diameter mycelial plugs were aseptically excised and transferred onto fresh Potato Dextrose Agar (PDA), Czapek Yeast Extract Agar (CYA), Malt Extract Agar (MEA) and oatmeal agar (OA) plates to assess colony morphology and growth performance, following the protocols of [44,45]. *Fusarium* cultures were incubated at 25 °C under dark conditions, and colony diameters were measured after five and seven days. Growth rate (mm day^−1^) was calculated based on the increase in colony diameter over time. For *Penicillium* isolates, colony characteristics and growth rates were evaluated on CYA and MEA. Macroscopic features, including colony color, texture, pigmentation, and exudation, were recorded and photographed. For microscopic characterization, the isolates were cultured on genus-specific media. *Fusarium* isolates were grown on carnation leaf agar (CLA) and synthetic nutrient-poor agar (SNA) and incubated at 25 °C under either a 12 h light/12 h dark photoperiod or continuous darkness to induce sporulation and morphological differentiation [44,46]. After 3–5 days, developing aerial mycelia, microconidia, and conidiogenous cells were examined and photographed. After 1–2 weeks, sporodochial conidia, aerial conidia, conidiogenous structures, and chlamydospores were observed and documented. *Penicillium* isolates were cultured on MEA for detailed microscopic examination of conidiophores and conidia.

Microscopic observations were performed using a compound light microscope (Nikon Eclipse 80i, Tokyo, Japan). Images were captured and analyzed with NIS-Elements BR software (version 3.2). Measurements of each morphological structure (e.g., *conidia*, *conidiogenous cells*, *chlamydospores*, *phialides*, *Metulae)* were obtained from at least 30 independent observations to ensure statistical reliability.

### 2.4. DNA Extraction and PCR Amplification

Genomic DNA was extracted from actively growing mycelia obtained from five-day-old cultures maintained on potato dextrose agar (PDA) at 25 °C. Approximately 100 mg of fresh mycelium was scraped from the colony surface using a sterile spatula and transferred into 1.5 mL microcentrifuge tubes. The DNA extraction was performed using the Fungal Genomic DNA Extraction Kit (Aidlab Biotechnologies Co., Beijing, China), following the manufacturer’s instructions. The quality and concentration of extracted DNA were verified by spectrophotometry (NanoDrop 2000, Thermo Fisher Scientific, Waltham, MA, USA) and agarose gel electrophoresis (1% agarose, 1× TAE buffer). High-quality DNA samples were stored at –20 °C until use. Six nuclear loci were targeted for amplification to support species-level identification and phylogenetic analyses: the internal transcribed spacer (ITS) region, translation elongation factor 1-alpha *(tef1-α*), calmodulin (*cmdA*), β-tubulin (*tub2*), and the RNA polymerase subunits I and II (*rpb1* and *rpb2*). Amplifications were performed using primer pairs ITS4/ITS5, EF1/EF2, CL1/CL2A, CMD5/CMD6, Bt2a/Bt2b, and fa/g2r, 5f2/7cr, respectively (Table 2). Each PCR reaction (25 µL total volume) contained 12.5 µL DreamTaq Green Master Mix (Thermo Fisher Scientific), 1 µL of each primer (10 µM), 2 µL genomic DNA (20–50 ng), and nuclease-free water to volume. The thermocycling conditions consist of 94–95 °C for 5 min; followed by 35 cycles of denaturation at 94–95 °C for 30–60 s, primer-specific annealing at 53–58 °C for 45–60 s, and extension at 72 °C for 1 min/kb; with a final extension at 72 °C for 10 min (Table 2). PCR products were separated by 1.5% agarose gels stained with SYBR Safe (Invitrogen, Tianyi Huiyuan Science and Technology Corporation Ltd., Guangzhou, China) and visualized under UV illumination. Amplicons were purified using ExoSAP-IT (Tianyi Huiyuan Science and Technology Corporation Ltd., Guangzhou, China) and sequenced bidirectionally by Sangon Biotech (Shanghai, China). All sequences generated were deposited in GenBank (Table 3 and Table 4) for reference in subsequent phylogenetic analyses.

**Table 1:** PCR amplification conditions were used in the thermal cycler for each gene.

Locus	Primer (Forward/Reverse)	PCR Conditions	References
ITS	ITS4/ITS5	94 °C 3 min, 35 cycles of 94 °C 30 s, 55 °C 50 s, 72 °C 1 min; 72 °C 10 min	[47]
*cmdA*	CMD5, CMD6 CL1/CL2A	95 °C 5 min, 35 cycles of 94 °C 45 s, 55 °C 45 s, 72 °C 1 min; 72 °C 10 min	[48,49]
*tef1-α*	EF1/EF2	95 °C 5 min, 40 cycles of 95 °C 1 min, 56 °C 1 min, 72 °C 1 min; 72 °C 10 min	[49]
*tub2*	Bt2a/Bt2b	94 °C 3 min, 35 cycles of 94 °C 30 s, 55 °C 50 s, 72 °C 1 min; 72 °C 10 min	[50]
*rpb2*	5f2/7cR 5f/7cR	95 °C 5 min, 40 cycles of 95 °C 1 min, 53 °C 1 min, 72 °C 1 min; 72 °C 10 min	[51]
*rpb1*	fa/g2r	95 °C 5 min, 40 cycles of 95 °C 1 min, 55 °C 1 min, 72 °C 1 min; 72 °C 10 min	[49,52]

**Table 2:** List of *Fusarium* and *Penicillium* strains isolated from Chili Peppers in Guangzhou during 2023–2025.

Strains	*Fusarium* and *Penicillium* Species	The Name of Markets	Year of Isolation
1. ZHKUCC 25-1342	*F. concentricum*	Jiangnan Market	2023
2. ZHKUCC 25-1343	*F. fujikuroi*	Jiangnan Market	2023
3. ZHKUCC 25-1344	*F. sulawesiense*	Xinfeng Market	2025
4. ZHKUCC 25-1345	*F. commune*	Xiao Gang Road Market	2023
5. ZHKUCC 25-1346, ZHKUCC 25-1347, ZHKUCC 25-1348, ZHKUCC 25-1349, ZHKUCC 25-1350	*F. verticillioides*	Shayuan Market, Jiangnan Market, Panlong Market	2024
6. ZHKUCC 25-1351, ZHKUCC 25-1352, ZHKUCC 25-1353, ZHKUCC 25-1354, ZHKUCC 25-1355	*F. annulatum*	Xiao Gang Road Market, Sha yuan, Panlong Market	2024
7. ZHKUCC 25-1356	*F. pernambucanum*	Lujiang Market	2024
8. ZHKUCC 25-1357	*F. ramsdenii*	Xiao Gang Road Market	2023
9. ZHKUCC 25-1358	*F. tardichlamydosporum*	Panlong Market	2023
10. ZHKUCC 25-1359	*F. compactum*	Xiao Gang Road Market	2024
11. ZHKUCC 25-1360, ZHKUCC 25-1361, ZHKUCC 25-1362, ZHKUCC 25-1363, ZHKUCC 25-1364, ZHKUCC 25-1365, ZHKUCC 25-1366, ZHKUCC 25-1367	*P. citrinum*	Xiao Gang Road Market, Panlong Market, Qingping Market	2024
12. ZHKUCC 25-1368	*P. steckii*	Xiao Gang Road Market	2024

### 2.5. Phylogenetic Analyses

Phylogenetic analyses were performed following the procedures described by Dissanayake [53]. Sequence data for all loci were initially compared against the NCBI database using BLASTnv. 2.16.0 Basic Local Alignment Search Tool; https://blast.ncbi.nlm.nih.gov/Blast.cgi (accessed on 21 Jun 2025) to retrieve the most closely related sequences from GenBank and recently published studies [54,55]. Individual loci were aligned using MAFFT version 7.036 http://mafft.cbrc.jp/alignment/server/large.html (accessed on 4 September 2025). Ref. [56] with default settings, and alignments were manually refined using BioEdit v.7.0.5.2 [57] prior to phylogenetic inference. Combined alignments in FASTA format were converted to PHYLIP and NEXUS formats via the online Alignment Transformation Environment ALTER; https://www.sing-group.org/ALTER (accessed on 28 September 2025) for Maximum Likelihood (ML) and Bayesian analyses, respectively. ML analyses were conducted using IQ-TREE on XSEDE v.2.3.2 (CIPRES Science Gateway platform) [58], applying the GTRGAMMA model of evolution and generating support values with 1000 ultrafast bootstrap replicates (IQ-TREE). Bayesian inference (BI) was carried out in MrBayes v.3.1.2 [59] using Markov Chain Monte Carlo (MCMC) sampling with four simultaneous chains, run for 1,000,000 generations, sampled every 100th generation. The first 25% of trees were discarded as burn-in, and posterior probabilities (PP) were calculated from the remaining 75% of trees. Phylogenetic trees were visualized in FigTree v.1.4.0 [60] and finalized using Adobe Illustrator CC 22.0.0 (Adobe Systems, San Jose, CA, USA).

### 2.6. Sample Preparation for Toxin Extraction

For mycotoxin analysis, 66 samples (33 fresh, 33 dried) representing 11 treatments (A–K) were selected. Each treatment corresponds to a specific market source. For each analysis, 5.0 g of a finely ground chili pepper sample was accurately weighed and transferred into a clean centrifuge tube. To the sample, 20 mL of extraction solvent consisting of acetonitrile, water, and formic acid (70:29:1, *v*/*v*/*v*) was added. The mixture was either shaken on a mechanical shaker for 1 h or homogenized at high speed for 2 min to ensure complete release of mycotoxins. The suspension was then centrifuged at 6000 rpm for 5 min, and the resulting supernatant was collected as the crude extract.

### 2.7. Mycotoxin Extraction from Penicillium and Fusarium Isolates

A subset of 23 strains was selected for targeted mycotoxin production assays based on their known or suspected toxigenic potential. Specifically, we focused on *Fusarium* species within the FFSC and FIESC complexes that are reported producers of fumonisins, particularly fumonisin B_1_ (FB_1_). Similarly, from the *Penicillium* isolates belonging to section *Citrina*, we selected species known for their capacity to produce citrinin. This targeted approach allowed us to directly evaluate the toxigenic potential of the most relevant fungal species for the two major toxin classes (fumonisins and citrinin) detected in our chili pepper survey, excluding species with no documented potential to produce these specific metabolites. Each selected *Fusarium* strain was cultured on PDA medium to assess FB_1_ production, and each selected *Penicillium* strain was cultured on both CYA and YES media to evaluate citrinin production under different nutritional conditions in the dark at 25 °C for 15 days. Mycotoxin extraction from both fungal groups was performed concurrently, with citrinin obtained from *Penicillium* grown on CYA and YES media, and fumonisin B_1_ extracted from *Fusarium* isolates cultivated on PDA. For each analysis, one gram of fungal culture was extracted with 5 mL of extraction solvent consisting of acetonitrile, water, and formic acid (70:29:1, *v*/*v*/*v*). The mixture was either shaken on a mechanical shaker for 1 h or homogenized at high speed for 2 min to ensure complete release of mycotoxins. The suspension was then centrifuged at 6000 rpm for 5 min, and the resulting supernatant was collected as the crude extract.

### 2.8. Purification

A 4 mL aliquot of the crude extract was carefully transferred into a non-fluorescent glass tube and purified by passing it through an MFC100 column (Qingdao Pribolab Biotech Co., Ltd., Qingdao, China) (Q), which effectively removed interfering matrix components. The eluate was collected as the purified extract. An appropriate volume of this solution was evaporated to dryness under a gentle stream of nitrogen gas, after which the residue was reconstituted in methanol. The resulting solution was used directly for instrumental analysis by liquid chromatography–mass spectrometry (LC–MS).

### 2.9. LC–MS/MS Analysis and Method Validation

Standard stock solutions (>99% purity) for DON, AFB_1_, OTA, FB_1_, ZEN, and citrinin were prepared in methanol. Matrix-matched calibration was employed using blank extracts of mycotoxin-free fresh and dried chili peppers spiked with standards across the working range. Chromatographic separation was used on a C18 column (50 mm × 2.1 mm, 2.7 μm) with mobile phase A (water with 1% acetic acid and 5 mM ammonium acetate) and B (methanol). The gradient: 5% B increased to 95% over 5 min, held 1 min, decreased to 10% in 0.1 min, and equilibrated 2 min. Flow rate: 0.3 mL/min; column temperature: 35 °C; injection volume: 2 μL. MS analysis was performed in MRM mode with nitrogen as the carrier gas, a curtain gas at 30 psi, a collision gas at 7 psi, nebulizing/auxiliary gases at 50 psi, and an ion source at 550 °C. MRM transitions were monitored for all six mycotoxins. Analytical standards were from (Qingdao Pribolab Biotech Co., Ltd., Qingdao, China), and solvents from Macklin. Reagent blanks, matrix blanks, and system suitability runs were routinely analyzed to monitor contamination and carry-over effects.

The method was validated for linearity, LOD, LOQ, recovery, matrix effect (ME), specificity, and precision. Recovery experiments used spiked blank samples at three levels: Recovery (%) = (observed/expected) × 100. LOD = 3 × σ/slope, LOQ = 10 × σ/slope, using the standard deviation of low-level spiked samples (σ) and calibration slope. ME (%) = (peak area in spiked matrix/peak area in solvent) × 100. Linearity (R^2^) was calculated using linear regression of peak area vs. concentration, with R^2^ ≥ 0.99 considered excellent. Specificity was confirmed by comparing retention times and MRM transitions with spiked samples.

### 2.10. Statistical Analysis

The experiment was conducted using a completely randomized design (CRD). Data was analyzed using SAS software (version 6.4). Three replicates were performed for each mycotoxin (DON, AFB_1_, OTA, FB_1_, ZEN, and CIT) in treatments (A–K), each treatment representing samples from a specific Guangzhou market. The concentration of each mycotoxin in each sample was measured in µg/g, and the mean and standard deviation (SD) were calculated for each treatment based on the three replicates. Relative standard deviation (RSD) was calculated as (SD/mean) × 100 to quantify variability among replicates. For overall reporting, the mean and SD of the treatment means (i.e., mean between treatments) were presented as Average ± SD. The lowest and highest treatment means were reported as Minimum and Maximum, respectively, and the SD for each was taken as the SD of the corresponding treatment (i.e., the SD of the replicates for that treatment). The percentage of contaminated samples was calculated as the proportion of samples with mycotoxin levels above zero relative to the total number of samples. All statistical calculations were performed in Excel. The confidence interval (CI) was calculated using the t-distribution as follows: CI = mean ± t (*n* − 1, 0.975) × (SD/√*n*). For non-normally distributed data, the interquartile range (IQR) was calculated as Q3 − Q1.

## 3. Results

### 3.1. Phylogenetic Analysis

Among fungal isolates from symptomatic chili peppers, *Fusarium* (58.7%) and *Penicillium* (30.3%) were predominant, while other genera accounted for 11.0%.

**Figure 1:**
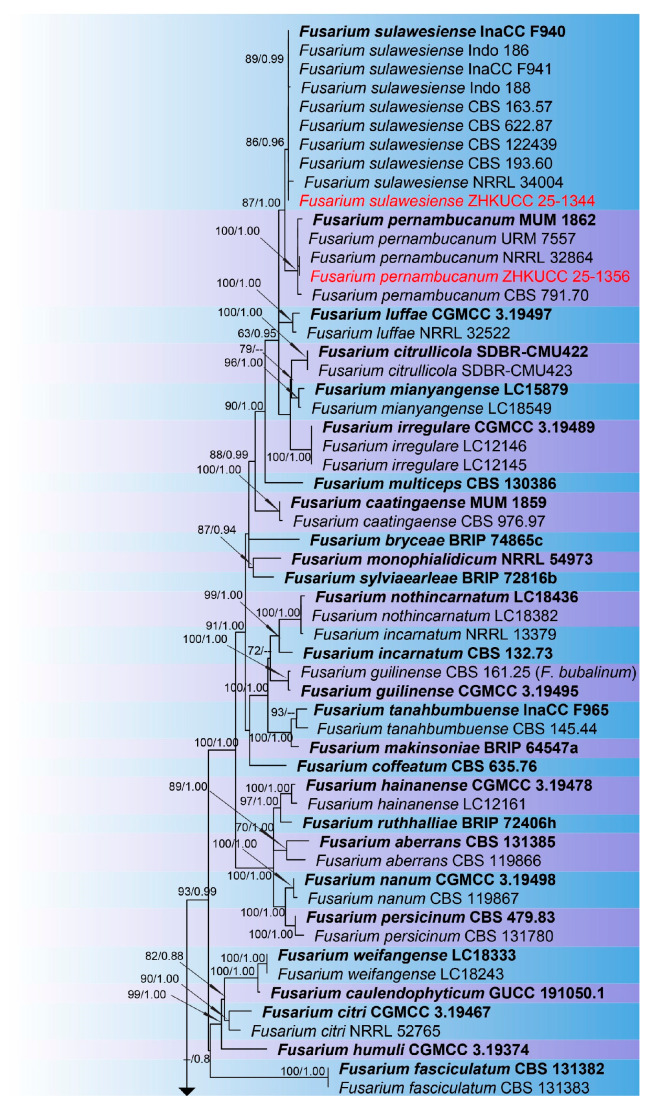

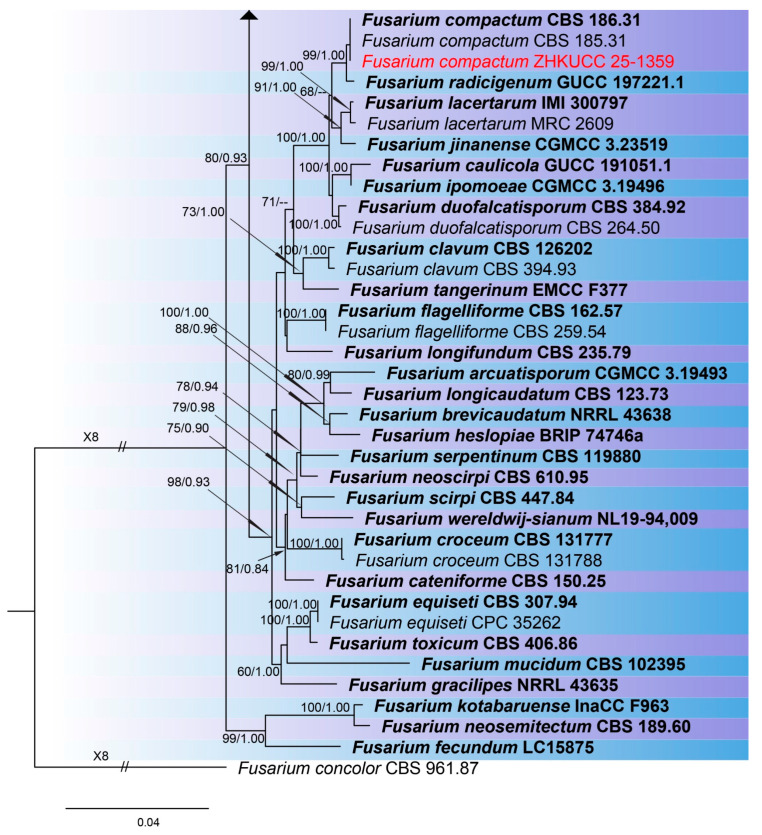
Maximum Likelihood phylogenetic tree inferred from the combined *cmdA, tef1-α, and rpb2* dataset of the *Fusarium incarnatum-equiseti* species complex (FIESC). ML bootstrap values ≥ 60% and Bayesian posterior probabilities (PP) ≥ 0.80 are shown at the nodes (ML/PP). The tree is rooted with *Fusarium concolor* (CBS 961.87). Isolates generated in this study are highlighted in red, and type or ex-type strains are presented in bold black. Phylogenetic analyses placed isolate ZHKUCC 25-1344 with the type strain InaCC F940 (86% ML, 0.96 PP) as *F. sulawesiense* and ZHKUCC 25-1356 with the type strain MUM 1862 (100% ML, 1.00 PP 1) as *F. pernambucanum*, ZHKUCC 25-1359 in a well-supported clade with *Fusarium compactum* (99% ML, 1.00 PP); (see Table 3 for GenBank accession numbers).

**Figure 2:**
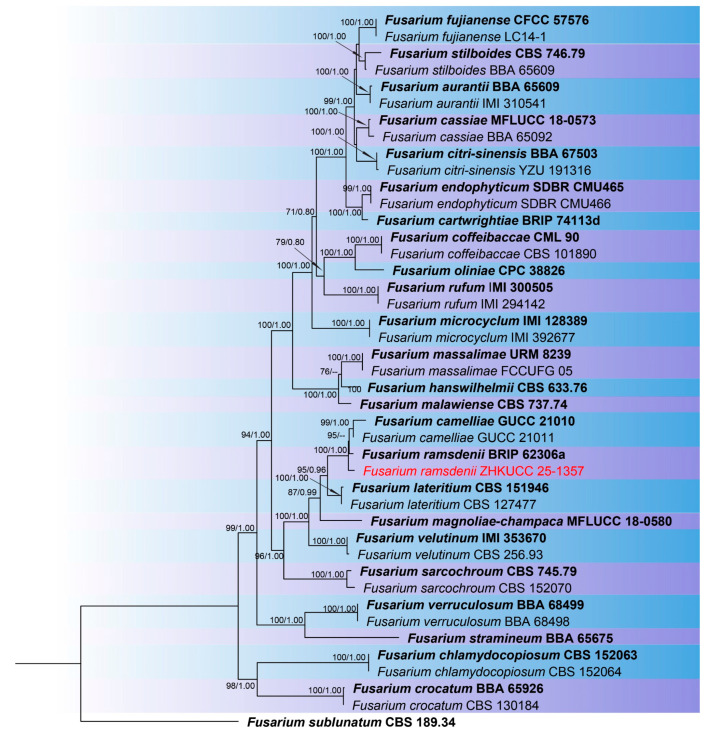
Maximum Likelihood phylogenetic tree inferred from the combined *cmdA*, *tef*1-α, *tub*2, and *rpb*2 dataset of the *Fusarium lateritium* species complex (FLSC). ML bootstrap values ≥ 60% and Bayesian posterior probabilities (PP) ≥ 0.80 are shown at the nodes (ML/PP). The tree is rooted with *Fusarium sublunatum* (CBS 189.34). Isolates generated in this study are highlighted in red, and type or ex-type strains are presented in bold black. Phylogenetic analyses placed isolate ZHKUCC 25-1357 with (100% ML, 1.00 PP), as *F. ramsdenii;* (see Table 3 for GenBank accession numbers).

**Figure 3:**
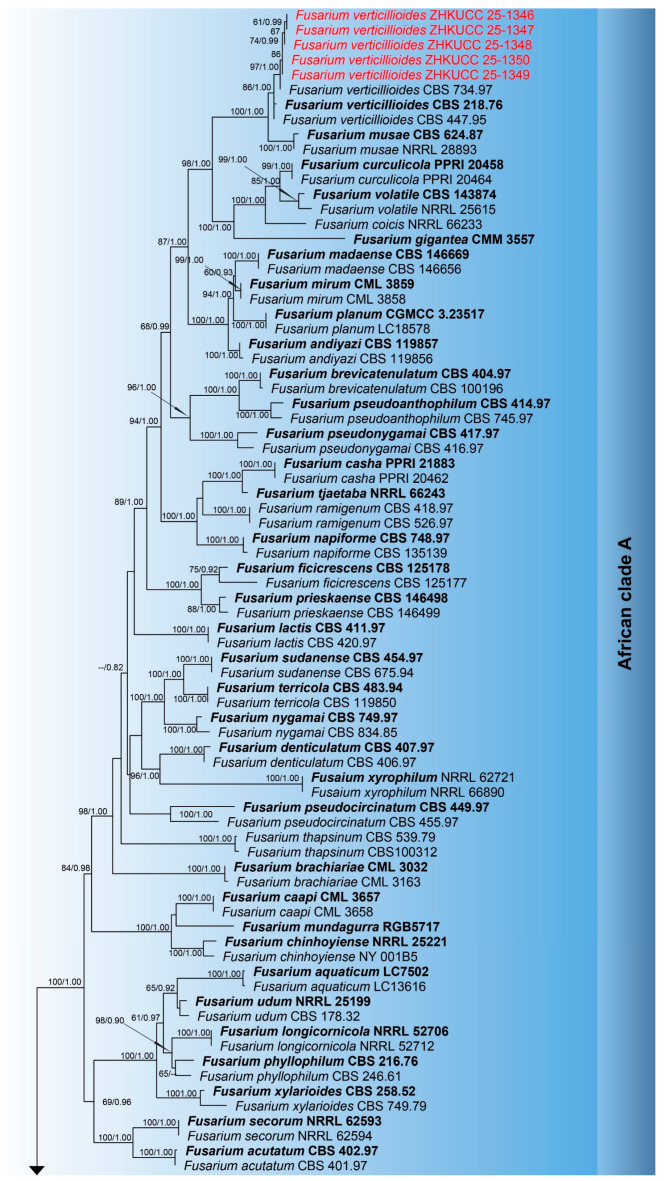

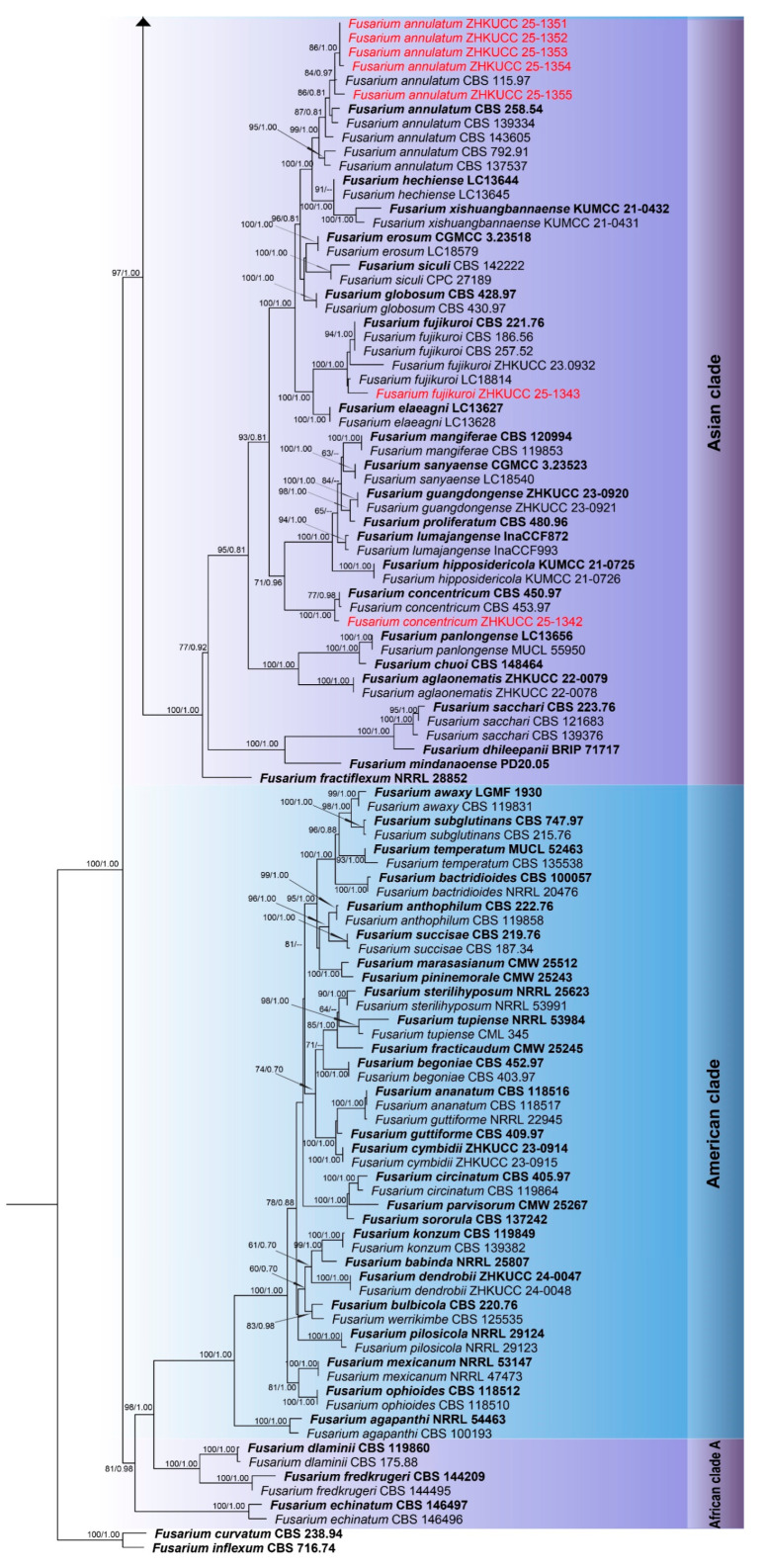
Maximum Likelihood phylogenetic tree inferred from the combined *cmdA*, *tef*1-α, *tub*2, *rpb*1, and *rpb*2 dataset of the *Fusarium fujikuroi* species complex (FFSC). ML bootstrap values ≥ 60% and Bayesian posterior probabilities (PP) ≥ 0.80 are shown at the nodes (ML/PP). The tree is rooted with *Fusarium curvatum* (CBS 238.94) and *F. inflexum* (CBS 716.74). Isolates generated in this study are highlighted in red, and type or ex-type strains are presented in bold black. Phylogenetic analyses placed five isolates (ZHKUCC 25-1346 to 25-1350) with *Fusarium verticillioides* (86% ML, 1.00 PP). Phylogenetic analyses placed isolates ZHKUCC 25-1351 to 25-1355 with type strain CBS 258.54 (99% ML, 1.00 PP), as *F. annulatum.* ZHKUCC 25-1343 with the ex-type strain CBS 221.76 (100% ML, 1.00 PP), as *F. fujikuroi*. ZHKUCC 25-1342 with *Fusarium concentricum* (CBS 450.97) (100% ML, 1.00 PP); (see Table 3 for GenBank accession numbers).

**Figure 4:**
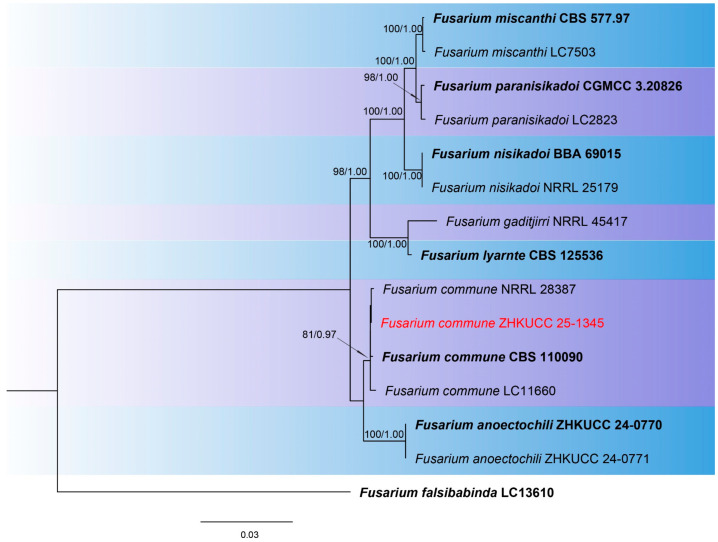
Maximum Likelihood phylogenetic tree inferred from the combined *tef1-α*, *tub2*, *rpb1*, and rpb2 dataset of the *Fusarium nisikadoi* species complex (FNSC). ML bootstrap values ≥ 60% and Bayesian posterior probabilities (PP) ≥ 0.80 are shown at the nodes (ML/PP). The tree is rooted with *Fusarium falsibabiinda* (LC13610). Isolates generated in this study are highlighted in red, and type or ex-type strains are presented in bold black. Phylogenetic analyses placed isolate ZHKUCC 25-1345 with *Fusarium commune* (81% ML, 0.97 PP); (see Table 3 for GenBank accession numbers).

**Figure 5:**
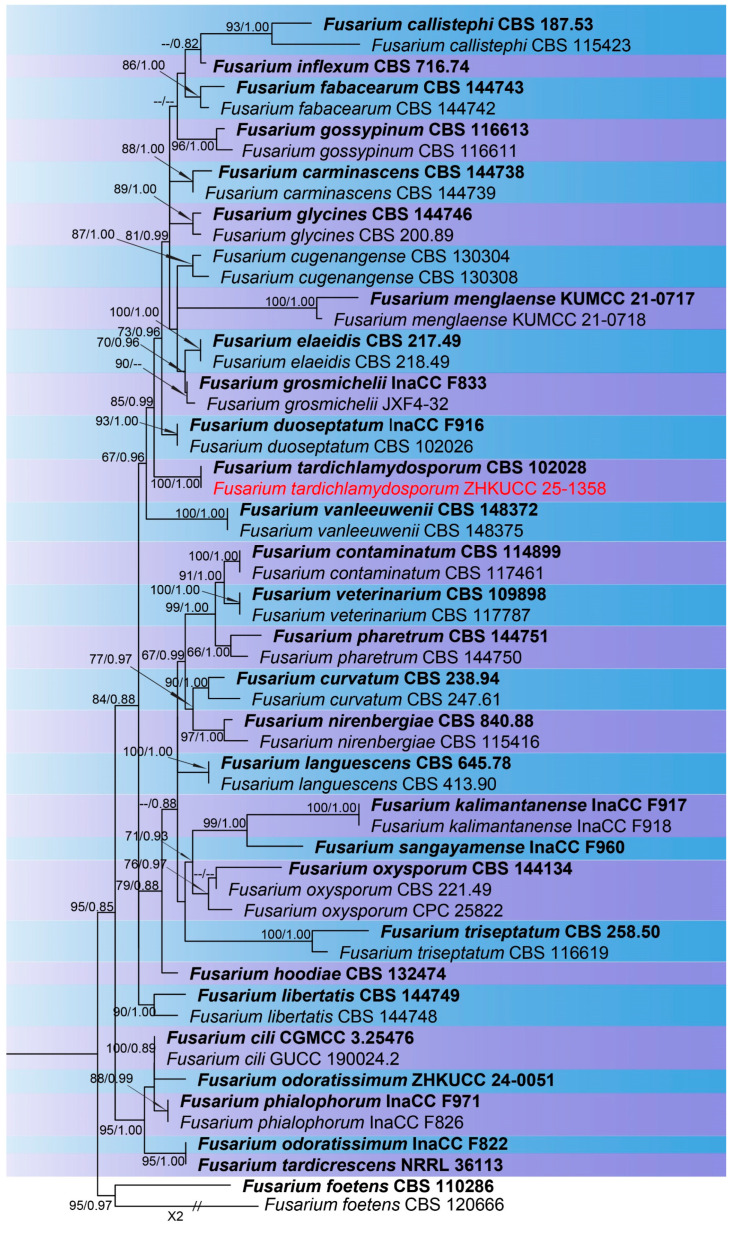
Maximum Likelihood phylogenetic tree inferred from the combined *cmdA*, *tef1-α*, *tub*2, and *rpb*2 dataset of the *Fusarium oxysporum* species complex (FOSC). ML bootstrap values ≥ 60% and Bayesian posterior probabilities (PP) ≥ 0.80 are shown at the nodes (ML/PP). The tree is rooted with *Fusarium fotens* (CBS 110286, CBS 120666). Isolates generated in this study are highlighted in red, and type or ex-type strains are presented in bold black. Phylogenetic analyses placed isolate ZHKUCC 25-1358 with *Fusarium tardichlamydosporum* (100% ML, 1.00 PP); (see Table 3 for GenBank accession numbers).

**Figure 6:**
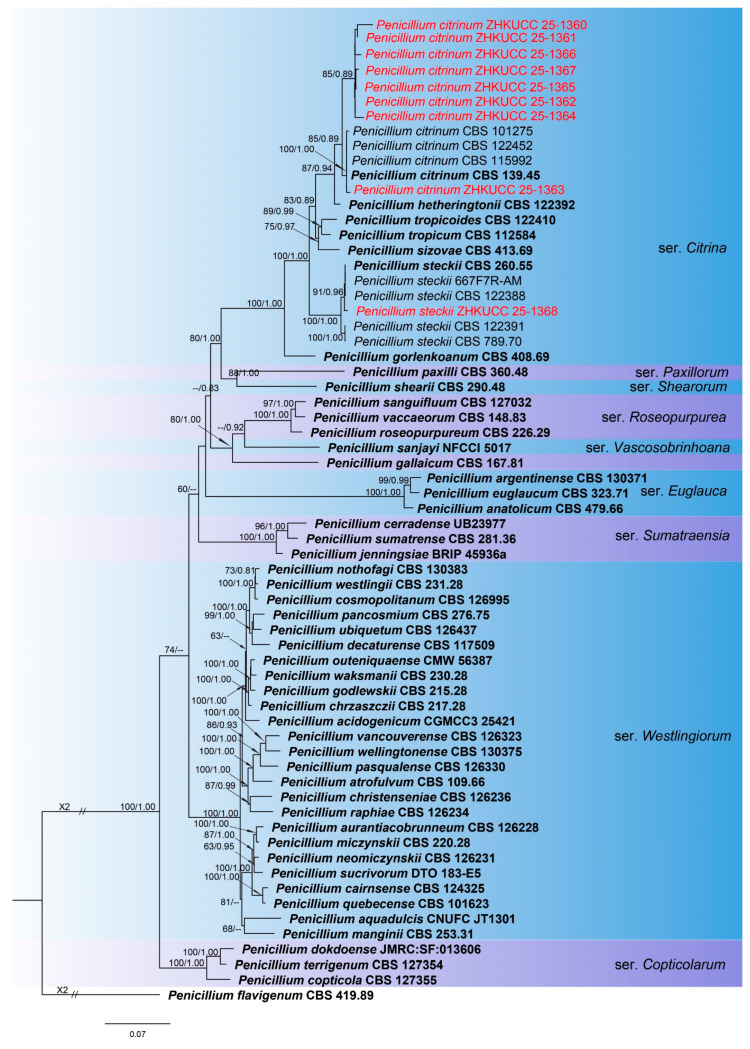
Maximum Likelihood phylogenetic tree inferred from the combined ITS, *tub2*, *cmdA*, and *rpb*2 dataset of the *Penicillium* section *Citrina*. ML bootstrap values ≥ 60% and Bayesian posterior probabilities (PP) ≥ 0.80 are shown at the nodes (ML/PP). The tree is rooted with *Penicillium flavigenum* (CBS 419.89). Isolates generated in this study are highlighted in red, and type or ex-type strains are presented in bold black. Phylogenetic analyses placed eight isolates (ZHKUCC 25-1360 to 25-1367) with *Penicillium citrinum* (85% ML, 0.89 PP), and ZHKUCC 25-1368 with the ex-type strain CBS 260.55 (100% ML, 1.00 PP) as *Penicillium steckii*; (see Table 4 for GenBank accession numbers).

- The combined phylogenetic analysis based on *cmdA*, *tef*1-α, and *rpb*2 sequences for *Fusarium incarnatum-equiseti* species complex (FIESC) isolates included 93 ingroup taxa, with *Fusarium concolor* (CBS 961.87) designated as an outgroup. The aligned dataset comprised 1981 characters: positions 1–549 for *cmdA*, 550–1387 for *rpb*2, 1388–1981 for *tef*1-α, including gaps. The best-scoring IQ-TREE yielded a final ML optimization likelihood value of −11,213.666, as shown in Figure 1. The dataset contained 655 distinct alignment patterns, 441 parsimony-informative sites, 264 singleton sites, and 1276 constant sites. The Bayesian analysis reached convergence at 1,000,000 generations, with an average standard deviation of split frequencies of 0.010457.
- The combined phylogenetic analysis based on *cmdA*, *tef*1-α, *tub2*, *rpb*1, and *rpb*2 sequences for *Fusarium fujikuroi* species complex (FFSC) isolates included 184 ingroup taxa, with *Fusarium curvatum* (CBS 238.94) and *F*. *inflexum* (CBS 716.74) designated as outgroups. The aligned dataset comprised 3979 characters: positions 1–524 for *cmdA*, 525–1154 for *tef*1-α, 1155–1614 for *tub2*, 1615–3213 for *rpb*1, and 3214–3979 for *rpb*2, including gaps. The best-scoring IQ-TREE yielded a final ML optimization likelihood value of −25,289.136, as shown in Figure 2. The dataset contained 1540 distinct alignment patterns, 1070 parsimony-informative sites, 169 singleton sites, and 2740 constant sites. The Bayesian analysis reached convergence at 1,000,000 generations, with an average standard deviation of split frequencies of 0.045165.
- The combined phylogenetic analysis based on *cmdA*, *tef*1-α, *tub2*, and *rpb*2 sequences for *Fusarium lateritium* species complex (FLSC) isolates included 42 ingroup taxa, with *Fusarium sublunatum* (CBS 189.34) designated as an outgroup. The aligned dataset comprised 2496 characters: positions 1–609 for *tef*1-α, 610–1105 for *tub2*, 1106–1942 for *rpb*2, and 1943–2496 for *cmdA*, including gaps. The best-scoring IQ-TREE yielded a final ML optimization likelihood value of −11,733.255, as shown in Figure 3. The dataset contained 868 distinct alignment patterns, 624 parsimony-informative sites, 205 singleton sites, and 1667 constant sites. The Bayesian analysis reached convergence at 1,000,000 generations, with an average standard deviation of split frequencies of 0.004700.
- The combined phylogenetic analysis based on *tef*1-α, *tub2*, *rpb*1, and *rpb*2 sequences for *Fusarium nisikadoi* species complex (FNSC) isolates included 14 ingroup taxa, with *Fusarium falsibabinda* (LC13610) designated as an outgroup. The aligned dataset comprised 3278 characters: positions 1–589 for *tef*1-α, 590–859 for *tub2*, 860–2437 for *rpb*1, and 2438–3278 for *rpb*2, including gaps. The best-scoring IQ-TREE yielded a final ML optimization likelihood value of −7450.967, as shown in Figure 4. The dataset contained 250 distinct alignment patterns, 167 parsimony-informative sites, 344 singleton sites, and 2767 constant sites. The Bayesian analysis reached convergence at 1,000,000 generations, with an average standard deviation of split frequencies of 0.006394.
- The combined phylogenetic analysis based on *cmdA*, *tef*1-α, *tub2*, and *rpb*2 sequences for *Fusarium oxysporum* species complex (FOSC) isolates included 55 ingroup taxa, with *Fusarium foetens* (CBS 110,286 and CBS 120,666) designated as outgroups. The aligned dataset comprised 2548 characters: positions 1–603 for *cmdA*, 604–1217 for *tef*1-α, 1218–1713 for *tub*2, and 1714–2548 for *rpb*2, including gaps. The best-scoring IQ-TREE yielded a final ML optimization likelihood value of −5838.608, as shown in Figure 5. The dataset contained 261 distinct alignment patterns, 140 parsimony-informative sites, 103 singleton sites, and 2305 constant sites. The Bayesian analysis reached convergence at 1,000,000 generations, with an average standard deviation of split frequencies of 0.007499.
- The combined phylogenetic analysis based on ITS, *tub2*, *cmdA*, and *rpb*2 sequences for *Penicillium* isolates from section *Citrina* included 64 ingroup taxa, with *Penicillium flavigenum* (CBS 419.89) designated as an outgroup. The aligned dataset comprised 2370 characters: positions 1–521 for ITS, 522–945 for *tub2*, 946–1459 for *cmdA*, and 1460–2370 for *rpb*2, including gaps. The best-scoring IQ-TREE yielded a final ML optimization likelihood value of −22,295.550, as shown in Figure 6. The dataset contained 1084 distinct alignment patterns, 852 parsimony-informative sites, 191 singleton sites, and 1521 constant sites. The Bayesian analysis reached convergence at 1,000,000 generations, with an average standard deviation of split frequencies of 0.045396.

### 3.2. Taxonomy

All symptomatic samples were subjected to fungal isolation, yielding fungal growth in 52.8% of cases. The isolates comprised *Fusarium* spp., *Penicillium* spp., and other fungal genera and a total of 12 fungal species were taxonomically determined by morphological characterization and multilocus phylogenetic analyses. A systematic literature review using PubMed, Google Scholar and USDA fungi databases confirmed whether these findings on chili pepper were novel by considering molecularly and morphologically confirmed identifications as definitive evidence of prior occurrence. This verification showed that seven species are being reported for the first time on *Capsicum annuum*.

#### 3.2.1. *Fusarium* Link, Mag. Gesell. Naturf. Freunde, Berlin 3 (1–2): 10 (1809)

##### *Fusarium fujikuroi* Species Complex (FFSC)

The FFSC includes important plant pathogens, mycotoxin producers, and occasional human opportunists globally [61,62,63,64]. Phylogenetic studies resolved the FFSC into African, American, and Asian clades [63,65,66]. In this study, 12 strains representing four species within the FFSC were identified:

*Fusarium annulatum*   Bugnic., Rev. gén. Bot. 59: 13 (1952)

Index Fungorum number: IF297536

Culture characteristics: Colonies on PDA 6.5 mm/day at 25 °C; white to pinkish violet. *Sporodochia* pale yellow on SNA. *Microconidia* clavate, 7–15.9 × 3.32–4.28 μm ($\bar{x}$ = 8 × 4.2, *n* = 30). *Macroconidia* 19.9–45.5 × 3.18–5 μm ($\bar{x}$ = 33 × 4.5, *n* = 30). *Chlamydospores* absent.

**Figure 7:**
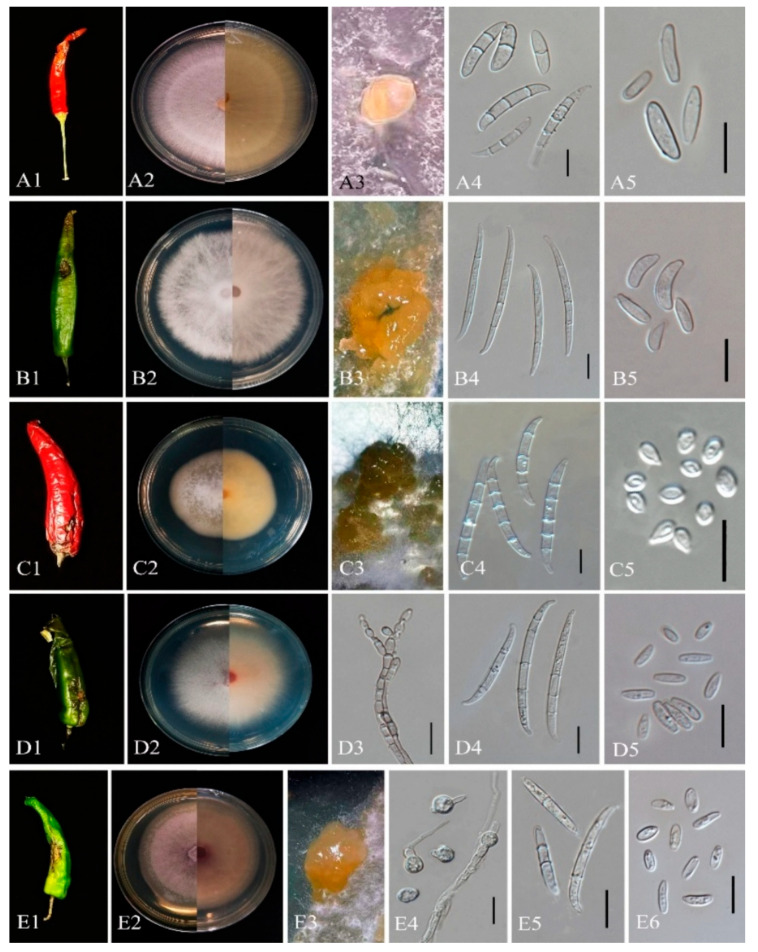
Morphological characteristics of five *Fusarium* species. *F*. *annulatum* (**A1**–**A5**), *F*. *concentricum* (**B1**–**B5**), *F*. *ramsdenii* (**C1**–**C5**), *F*. *verticillioides* (**D1**–**D5**), *F*. *tardichlamydosporum* (**E1**–**E6**). Symptoms on hosts (**A1**,**B1**,**C1**,**D1**,**E1**); Colonies on PDA above and below (**A2**,**B2**,**C2**,**D2**,**E2**); Sporodochia on SNA (**A3**,**B3**,**C3**,**E3**); Macroconidia on SNA and CLA (**A4**,**B4**,**C4**,**D4**,**E5**); Microconidia (**A5**,**B5**,**C5**,**D5**,**E6**); Microconidia in chain (**D3**); Chlamydospores on SNA (**E4**). Scale bars = 10 µm.

Note: Strains ZHKUCC 25-1351–1355 with ex-type CBS 258.54 (99% ML, 1.00 PP; Figure 3), confirmed as *F. annulatum* (Figure 7).

*Fusarium concentricum* Nirenberg & O’Donnell, Mycologia 90 (3): 442 (1998) [67]

Index Fungorum number: IF444884

Culture characteristics: Colonies on PDA: 6 mm/day at 25 °C; surface reddish white; reverse white with pale zones. *Sporodochia* pale orange on SNA. *Microconidia* obovoid to allantoid, 7.8–10 × 3.2–3.9 μm ($\bar{x}$ = 8.5 × 3.5 μm, *n* = 30). *Macroconidia* 3-septate 39–53 × 2.7–3.5 μm ($\bar{x}$ = 45 × 3.5 μm, *n* = 30); 5-septate 51.4–63 × 3.5–4.3 μm ($\bar{x}$ = 55 × 4 μm, *n* = 30). *Chlamydospores* absent.

Note: Isolate ZHKUCC 25-1342 grouped with type strain CBS 450.97 (100% ML, 1.00 PP; Figure 3), confirmed as *F. concentricum* (Figure 7).

*Fusarium fujikuroi*  Mitt. biol. BundAnst.  Ld- u. Forstw. 169: 32 (1976)

Index Fungorum number: IF314213 Culture characteristics: Colonies on PDA: 6 mm/day at 25 °C; cottony, white to yellow orange; reverse zonate. *Sporodochia* on SNA. *Microconidia* 5.1–11.95 × 1.93–3.19 μm ($\bar{x}$ = 8 × 2.5 μm, *n* = 30). *Macroconidia* 27–47 × 2.5–4.5 μm ($\bar{x}$ = 38 × 3.5 μm, *n* = 30). *Chlamydospores* absent.

**Figure 8:**
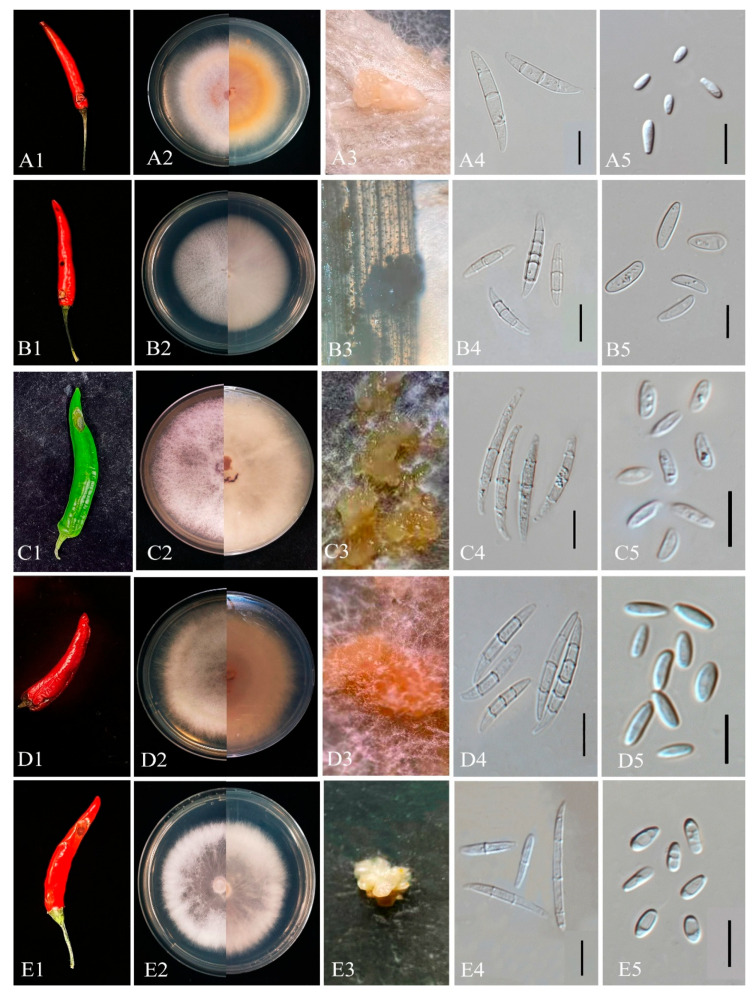
Morphological characteristics of five *Fusarium* species. *F*. *pernambucanum* **(A1–A5)**, *F*. *compactum* (**B1**–**B5**), *F*. *commune* (**C1**–**C5**), *F*. *sulawesiense* (**D1**–**D5**), *F*. *fujikuroi* (**E1**–**E5**). Symptoms on hosts (**A1**,**B1**,**C1**,**D1**,**E1**); Colonies on PDA above and below (**A2**,**B2**,**C2**,**D2**,**E2**); Sporodochia on SNA (**A3**,**B3**,**C3**,**D3**,**E3**); Macroconidia on CLA and SNA (**A4**,**B4**,**C4**,**D4**,**E4**); Microconidia (**A5**,**B5**,**C5**,**D5**,**E5**). Scale bars = 10 µm.

Note: Isolate ZHKUCC 25-1343 grouped with ex-type CBS 221.76 (100% ML, 1.00 PP; Figure 3), confirmed as *F. fujikuroi* (Figure 8).

*Fusarium verticillioides* (Sacc.) Nirenberg, Mitt. biol. BundAnst. Ld-u. Fo stw. 169: 26 (1976).

Index Fungorum number: IF314223

Culture characteristics: Colonies on PDA: 5 mm/day at 25 °C; white to pink-violet. *Sporodochia* absent. *Microconidia* in chains, 4–8 × 2–4 μm ($\bar{x}$ = 5 × 3.2, *n* = 30). *Macroconidia* rare, 25–55 × 3–5 μm ($\bar{x}$ = 42 × 4, *n* = 30). *Chlamydospores* absent.

Note: Five isolates (ZHKUCC 25-1346 to 25-1350) grouped with type strain *F. verticillioides* (86% ML, 1.00 PP; Figure 3), confirmed by morphology (Figure 7).

##### *Fusarium incarnatum-equiseti* Species Complex (FIESC)

*Fusarium incarnatum-equiseti* species complex (FIESC) comprises Cosmopolitan; species boundaries recently clarified [46,68,69,70]; produce trichothecenes, zearalenone [71,72]; some human pathogens [68,73]. Three species were identified in this complex.

*Fusarium compactum* (Wollenw.) Raillo, Fungi of the Genus Fusarium: 180 (1950)

IF297537

Culture characteristics: Colonies on PDA: 4.5 mm/day at 25 °C; yellowish white. *Sporodochia* translucent on CLA. *Microconidia* oval, 5.3–13.5 × 3.1–3.7 μm ($\bar{x}$ = 6.5 × 3, *n* = 30). Macroconidia curved, 18.3–62.5 × 2.8–5.6 μm ($\bar{x}$ = 48 × 3.2, *n* = 30). *Chlamydospores* absent.

Note: ZHKUCC 25-1359, grouped with type strain *F. compactum* (99% ML, 1.00 PP; Figure 1), confirmed by morphology (Figure 8).

*Fusarium pernambucanum*  A.C.S. Santos, C.S. Lima, P.V. Tiago & N.T. Oliveira

Index Fungorum number: IF825273

Culture characteristics: Colonies on PDA: 7 mm/day at 25 °C; rosy buff to hazel. *Sporodochia* on SNA. *Microconidia* fusoid, 4–10 × 2–4 μm ($\bar{x}$ = 7 × 3.2, *n* = 30). *Macroconidia* rare, 25–45 × 2.8–5.5 μm ($\bar{x}$ = 35 × 3.5, *n* = 30). *Chlamydospores* absent.

Note: ZHKUCC 25-1356, grouped with type strain MUM 1862 (100% ML, 1.00 PP; Figure 1), confirmed as *F. pernambucanum* (Figure 8).

*Fusarium sulawesiense*  Maryani, Sand. -Den., L. Lombard, Kema & Crous

Index Fungorum number: IF830777

Culture characteristics: Colonies on PDA: 6.5 mm/day at 25 °C; cottony, white to pale brown. Sporodochia pale orange on CLA. Microconidia fusoid, 3.5–10.5 × 2.5–4.5 μm ($\bar{x}$ = 8.5 × 3.58, *n* = 30). *Macroconidia* falcate, 30–42 × 2.5–4.5 μm ($\bar{x}$ = 35 × 3.7, *n* = 30). Chlamydospores absent.

Note: ZHKUCC 25-1344 grouped with type strain InaCC F940 (86% ML, 0.96 PP; Figure 1), confirmed (Figure 8).

##### *Fusarium lateritium* Species Complex (FLSC)

The *Fusarium lateritium* species complex (FLSC) was formally established in a phylogenetic study that incorporated *F. lateritium*, *F. stilboides*, and *F. sarcochroum* [74]. Species associated with arboreal hosts [75,76,77,78,79]. Mycotoxin production is poorly known [80]. One species was identified in this comples.

*Fusarium ramsdenii*  Y.P. Tan, Pegg, A.G. Manners, A.W. Cooke & R.G. Shivas, in Crous et al.

Index Fungorum number: IF843258

Culture characteristics: Colonies on PDA: 3.5 mm/day at 25 °C; pale violet. *Sporodochia* cream on SNA. *Microconidia* elliptical, 3.5–13.5 × 2.5–3.5 μm ($\bar{x}$ = 9.5 × 3, *n* = 30). *Macroconidia* straight to curved, 32–43 × 2.8–3.2 μm ($\bar{x}$ = 35 × 3, *n* = 30). *Chlamydospores* absent.

Note: ZHKUCC 25-1357 grouped with type strain *F. ramsdenii* (100% ML, 1.00 PP; Figure 2), confirmed (Figure 7).

##### *Fusarium nisikadoi* Species Complex (FNSC)

The *Fusarium nisikadoi* species complex (FNSC) currently comprises seven plant-associated species [44,55,81]. Several of which are recognized as pathogens of important crops such as rice and maize [82,83,84]. Although information on their secondary metabolites remains limited, there is evidence that some members, including *F. commune*, can produce mycotoxins such as beauvericin [85].

*Fusarium commune*  K.Skovg., O’Donnell & Nirenberg, in Skovgaard, Rosendahl, O’Donnell & Nirenberg.

Index Fungorum number: IF489435

Culture characteristics: Colonies on PDA 6 mm/day at 25 °C; white to pale pink. *Sporodochia* on SNA. *Microconidia* 5.5–7.5 × 2–3.5 μm ($\bar{x}$ = 6 × 3, *n* = 30). *Macroconidia* fusiform, 34–45 × 2.5–3.7 μm ($\bar{x}$ = 37 × 3, *n* = 30). *Chlamydospores* absent.

Note: ZHKUCC 25-1345 with type strain *F. commune* (81% ML, 0.97 PP; Figure 4), confirmed (Figure 8).

##### *Fusarium oxysporum* Species Complex (FOSC)

The *Fusarium oxysporum* species complex (FOSC) FOSC comprises soil-borne fungi causing wilts [86,87,88,89,90]; includes plant and human pathogens, morphologically indistinguishable [68,91,92]; some produce beauvericin and fusaric acid [93,94]. One species in this complex was identified.

*Fusarium tardichlamydosporum*  Maryani, L. Lombard, Kema & Crous, in Maryani, Lombard, Poerba, Subandiyah, Crous & Kema.

Index Fungorum number: IF826805

Culture characteristics: Colonies on PDA: 5.5 mm/day at 25 °C; surface purple centrally with white margins; sporodochia on CLA. *Sporodochia* on SNA. *Microconidia* ovoid to ellipsoid, 5.4–8.5 × 2.3–5.5 μm ($\bar{x}$ = 7 × 3.5 μm, *n* = 30). *Macroconidia* falcate, 38–45 × 5.3–6.8 μm ($\bar{x}$ = 42 × 5.5 μm, *n* = 30). *Chlamydospores* globose, rough-walled, 6.8–9.5 × 5.5–9 μm ($\bar{x}$ = 8 × 6.5 μm, *n* = 30), after 4 weeks.

Note: Isolate ZHKUCC 25-1358 grouped with *F. tardichlamydosporum* (100% ML, 1.00 PP; Figure 5), confirmed by morphology (Figure 7).

#### 3.2.2. *Penicillium* Link, Mag. Gesell. Naturf. Freunde, Berlin 3 (1-2): 16 (1809)

Index Fungorum number: IF9257

##### *Penicillium* Section *Citrina*

Widely distributed in soil, indoor environments, food products; occurrence linked to climate [95,96,97]. Characterized by symmetrically biverticillate conidiophores, small globose to subglobose conidia [98,99]. Produce citrinin and citreoviridin, varying by species [97,100,101]. Nine strains identified: eight *P. citrinum*, one *P. steckii.*

*Penicillium steckii*  K.W. Zaleski, Bull.  Acad. Polon. Sci., Math. et Nat., Sér. B: 469 (1927)

Index Fungorum number: IF278769

Culture characteristics: Colonies on CYA: 24–32 mm at 25 °C; MEA: 21–30 mm at 25 °C. *Sporulation* moderate to good; greyish green conidia; clear to pale yellow exudates; no soluble pigments.

Morphology: *Conidiophores* symmetrically biverticillate; stipes smooth, 2–3.3 µm wide. *Metulae* 13.5–18.8 × 2.5–3.2 µm. *Phialides* ampulliform, 6.8–10.5 × 2.2–3 µm. Conidia smooth, broadly ellipsoidal to slightly fusiform, 2.3–3.1 × 1.7–2.6 µm.

**Figure 9:**
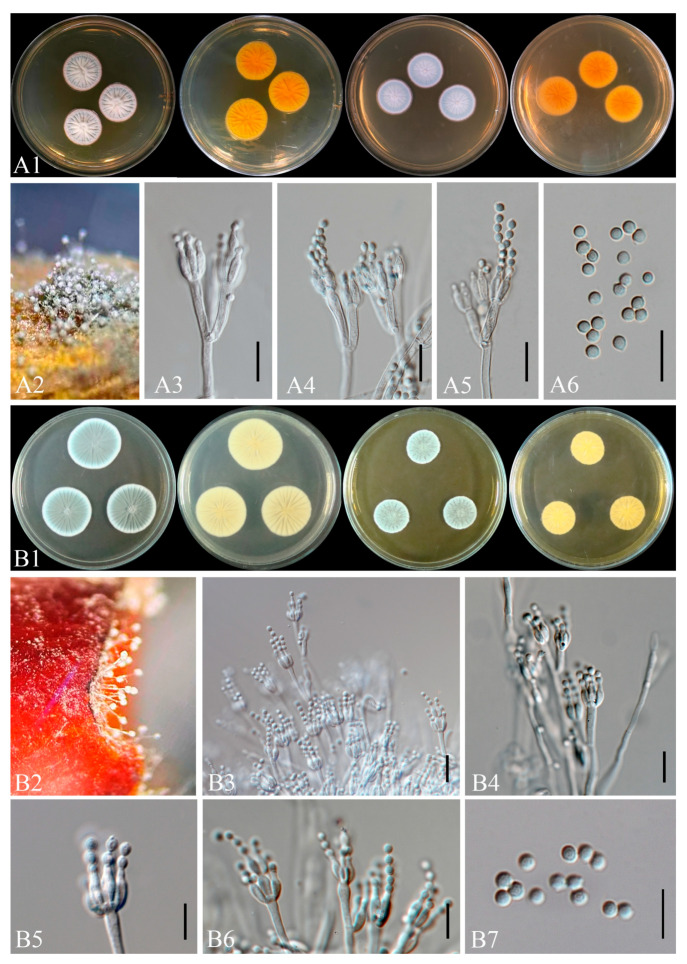
Morphological characteristics of two *Penicillium* species. *P. steckii* (**A1**–**A5**), *P. citrinum* (**B1**–**B7**); Colonies on CYA above and below (**A1 left**, **B1 left**), on MEA above and below (**A1 right**, **B1 right**); Conidiophores on host (**A2**, **B2**); Conidiophores and conidia of *P. steckii* (**A3**–**A5**), and conidiophores and conidia of *P. citrinum* (**B2**–**B7**). Scale bars = 10 µm.

Note: ZHKUCC 25-1368 grouped with ex-type CBS 260.55 (100% ML, 1.00 PP; Figure 9); morphology confirmed as *P. steckii* (Figure 9).

*Penicillium citrinum*  Thom, Bull. U.S. Department of Agriculture, Bureau of Animal Industry 118: 61 (1910).

Index Fungorum number: IF165293

Culture characteristics: Colonies on CYA: 27–33 mm at 25 °C; MEA: 18–25 mm at 25 °C. *Sporulation* moderate; greyish green to bluish *conidia*; pale yellow exudates; yellow diffusible pigments.

Morphology: *Conidiophores* mostly biverticillate; stipes smooth, 2–3 µm wide. Metulae 11.5–16.5 × 2–2.5 µm. *Phialides* ampulliform, 7.5–10 × 2–2.5 µm. *Conidia* smooth, globose to subglobose, 2–2.7 × 1.8–2.7 µm.

Note: Eight isolates (ZHKUCC 25-1360–1367) clustered with *P. citrinum* (85% ML, 0.89 PP; Figure 6); morphology matched key features (Figure 9), confirming identity.

### 3.3. Mycotoxin Production Profile

For the mycotoxin analysis, 66 samples (33 dried and 33 fresh) were tested in a single LC-MS/MS run for quantification of all mycotoxins, and calculated data on contamination are presented in Table 5 and Table 6. Statistical comparisons of both fresh and dried samples. Concentrations of six mycotoxins were analyzed in fresh and dried pepper samples across 11 treatments (A–K), each treatment representing a specific Guangzhou market. (Table 5 and Table 6). The six mycotoxins studied included DON, AFB_1_, OTA, FB_1_, ZEN, and citrinin. DON was detected at the highest average level in dried pepper samples at 0.56 µg/g, compared to 0.067 µg/g for FB_1_. At the same time, AFB_1_ was detected in only 9.09% of fresh samples and at concentrations below the LOQ. OTA was not detected in fresh samples. Citrinin was found in both matrices (fresh and dried), but at a higher average concentration of 0.039 µg/g in dried than in fresh of 0.019 µg/g. ZEN was produced at much lower levels but at a higher prevalence in dried (63.63%) than in fresh (27.27%).

DON concentration levels were significantly different between fresh and dried samples. DON was found at 0.087 µg/g in fresh peppers, with a range of variability of ±0.68 µg/g, and 63.63% of the samples were positive for DON. Dried peppers had a mean DON concentration of 0.56 µg/g, with a maximum of 1.74 µg/g and a 100% occurrence rate. Therefore, the results suggest that drying of peppers increases the concentration of DON and, correspondingly, higher toxin concentrations in dried than in fresh samples.

AFB_1_ was rarely detected in fresh samples (9.09% of samples, all below LOQ) but was detected in all dried samples (100%). AFB_1_ concentration levels in the dried samples were all above LOQ (mean concentration 0.025 µg/g).

The presence of OTA was not detected in fresh samples, but it was detected in dried samples at a mean concentration of 0.099 µg/g.

FB_1_ was detected in 72.72% of fresh samples at very low average concentrations (0.004 µg/g), with a maximum of 0.013 µg/g. Dried pepper supported 100% prevalence and increased FB_1_ levels, averaging 0.067 µg/g and reaching a maximum of 0.192 µg/g. These results suggest that the fumonisin B_1_ concentration was higher during drying.

ZEN production was limited across isolates under both conditions. Fresh pepper showed 27.27% prevalence with trace amounts (average 0.0006 µg/g, max 0.0044 µg/g). Dried pepper showed higher prevalence (63.63%) and slightly elevated average concentrations (0.003 µg/g, max 0.012 µg/g). Thus, ZEN production was minimal overall but increased in dried pepper.

Citrinin was detected in 100% of samples in both matrices. However, average concentrations were higher in dried peppers (0.039 µg/g, max 0.059 µg/g) compared to fresh peppers (0.019 µg/g, max 0.035 µg/g). These results indicate that citrinin concentrations were maintained under both conditions, but were higher after drying.

Across all six mycotoxins, dried pepper samples showed higher prevalence and concentrations for DON, AFB_1_, OTA, FB_1_, ZEN, and citrinin. The drying process was associated with elevated toxin accumulation across nearly all toxin classes, with DON and FB_1_ showing the most pronounced increases in both frequency and concentration.

The results of statistical analysis of 33 dried chili samples and 33 fresh chili samples (11 treatments with 3 replicates) showed that mycotoxin contamination was common in dried chili samples. Four mycotoxins, DON, AFB_1_, OTA, and FB_1_, were detected in 100% of the samples, followed by CIT and ZEN with prevalence rates of 81.81% and 63.63%, respectively. DON contamination was the highest, with a mean concentration of 0.56 ± 0.51 μg/g and a maximum of 1.74 μg/g, indicating the highest toxicity across the entire data set and underscoring the need for strict control of the drying and storage processes.

While OTA was very evenly distributed at low concentrations in dried samples, DON and FB_1_ showed the greatest concentration variability (Table 5), complicating risk management in these products. In the study of mycotoxin contamination of fresh pepper samples, the contamination pattern showed a significant difference from dried samples, so the mycotoxin CIT was identified as the widespread contaminant with a prevalence rate of 100% (in all 33 samples), and its average concentration was recorded as 0.019 ± 0.009 μg/g. This was followed by FB_1_, with a prevalence of 72.72%, and DON, with 63.63%. DON, with a mean of 0.087 ± 0.197 μg/g, still had the highest average concentration among the mycotoxins in the wet samples, but it was significantly lower than that observed in the dried samples. It is worth noting that OTA was not detected at all in wet samples, whereas AFB_1_, with a prevalence of only 9.09%, was present but below the limit of quantification (Table 6).

The precision of mycotoxin determination in fresh pepper samples was evaluated using STD and RSD. In comparing dry and fresh samples, dry peppers exhibited higher analyte concentrations and lower RSD values, indicating better repeatability and analytical precision. In dried samples, DON, OTA, ZEN, and CIT showed low variability, while AFB_1_ and FB_1_ displayed slightly higher RSDs in some samples, likely due to sample heterogeneity or higher analyte levels (Table 7). In contrast, fresh samples contained mostly undetectable levels of AFB_1_, OTA, and ZEN, with low variability, whereas FB_1_ and CIT showed relatively higher RSDs (up to 17.98%) due to low analyte concentrations, wet-matrix effects, and sample heterogeneity (Table 8). These results indicate that drying concentrates analytes and improves analytical precision, whereas fresh matrices pose additional analytical challenges, underscoring the need for method optimization to determine mycotoxins in high-moisture samples accurately. Furthermore, fresh pepper samples showed lower mycotoxin contamination levels compared to dried samples.

**Figure 10:**
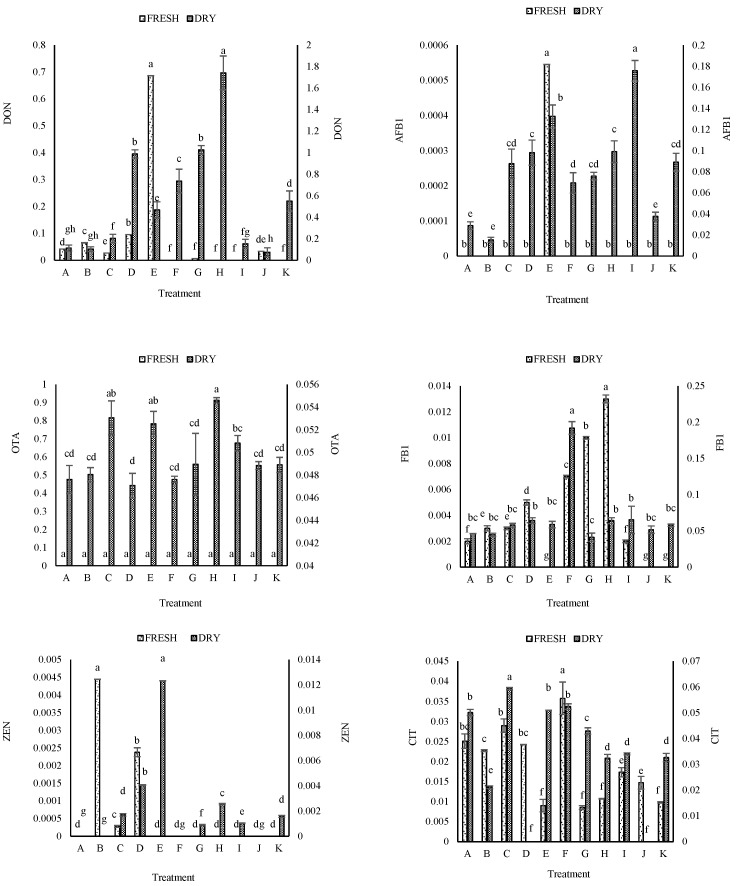
Comparison of mean concentrations of deoxynivalenol (DON), aflatoxin B_1_ (AFB_1_), ochratoxin A (OTA), fumonisin B_1_ (FB_1_), zearalenone (ZEN), and citrinin (CIT) in fresh and dried chili samples under different treatments (A–K). Treatments correspond to market sources. Different lowercase letters above the bars indicate statistically significant differences between treatments (*p* < 0.05). Bars sharing the same letter are not significantly different.

Based on the results of the analysis of variance, different treatments (A–K) had a significant effect on the mycotoxin contents of DON, AFB_1_, OTA, FB_1_, ZEN, and CIT (*p* < 0.01) in dried (Table 9) and fresh chili (Table 10). The results of the investigation into mycotoxin levels in different pepper samples showed that the concentration of these toxins in dried samples was significantly higher than in fresh samples. In dried samples, treatment H showed the highest level of contamination, especially for DON (1.741 µg/g), whereas in fresh samples, the levels of all toxins were very low or undetectable. The increase in mycotoxin concentrations in dried peppers is likely due to favorable conditions for fungal growth during drying and storage, including reduced humidity, elevated temperature, and inadequate ventilation. Among the toxins examined, DON and FB_1_ were most abundant in dried samples. In contrast, in fresh samples, almost all mycotoxins, including AFB_1_, OTA, and ZEN, were at negligible or zero levels. In contrast, CIT remained present, although at a lower concentration than in dried samples (Table 5 and Figure 10). A direct comparison between the two sample types suggests that drying may play an important role in increasing the risk of fungal contamination and mycotoxin production in peppers. The collective data suggest that the drying process is the critical factor creating favorable conditions for fungal proliferation and subsequent mycotoxin accumulation, thereby significantly elevating the contamination risk in dried chilies compared to their fresh counterparts.

A comparison of the validation parameters for the analysis of mycotoxins in dried and fresh chili (Table 11 and Table 12) revealed that the method performs with higher accuracy and reliability in dried chili. In dried chili (Table 8), recovery values for all mycotoxins were close to 100%, and matrix effects were generally low, which fulfils the requirements of Regulation (EC) No. 401/2006. In contrast, in fresh chili (Table 9), recovery was lower for some mycotoxins, particularly AFB_1_ (43.29%), and the matrix effect was pronounced for CIT (88.3%). Limits of detection (LOD) and quantification (LOQ) were low for both sample types, indicating adequate sensitivity; however, the quantification accuracy for some mycotoxins in fresh chili was limited. The coefficients of determination (R^2^) were high in both matrices, confirming the linearity of the calibration curves. Additionally, OTA was not detectable in fresh chili. Overall, the results indicate that the method is reliable and accurate for mycotoxin analysis in dried chili, while in fresh chili, adjustments or optimization are required for certain mycotoxins, particularly AFB_1_ and CIT.

AFB_1_ and CIT were often present at concentrations close to their LOQs (0.0074 and 0.0055 μg/g, respectively). This means that, despite acceptable sensitivity, small variations in extraction or matrix effects could have a relatively larger influence on quantification at these low levels. Therefore, additional replicates and/or confirmatory methods are recommended when AFB_1_ and CIT are detected near the LOQ.

### 3.4. Mycotoxin Production in Culture Media

On PDA medium, fumonisins (FB_1_) production was observed among selected *Fusarium* species (Table 13). *Fusarium verticillioides* showed positive FB_1_ production in 5 strains, with recorded values ranging from 0 to 4.62 µg/mL and a mean concentration of 1.924 µg/mL. Similarly, *F. annulatum* also had 5 positive strains, though toxin production was considerably lower, ranging from 0 to 0.34 µg/mL with an average value of 0.104 µg/mL. Other species showed only trace or no detectable FB_1_ production. *F. fujikuroi* produced a low concentration (0.13 µg/mL), while ***F. ramsdenii*** and *F. compactum* exhibited minimal FB_1_ levels of 0.16 µg/mL and 0.06 µg/mL, respectively. In contrast, *F. concentricum* showed no detectable FB_1_ production on PDA. These findings indicate that *F. verticillioides* was the most potent FB_1_ producer among the *Fusarium* species tested on PDA medium. Penicillium species produce citrinin (CIT) in varying amounts depending on the culture medium. On YES medium (Table 3). *Penicillium citrinum* produced significant amounts of citrinin, with eight positive strains detected. Concentrations ranged from 5.16 to 303 µg/mL, with a mean value of 220.08 µg/mL, indicating strong toxigenic potential under YES conditions. Additionally, *P. steckii* produced a mean citrinin level of 108.40 µg/mL on YES medium. On CYA medium, *P. citrinum* again demonstrated citrinin production in 8 strains, though at comparatively lower levels, ranging from 0 to 251.1 µg/mL, with an average concentration of 122.81 µg/mL. *Penicillium steckii* also produced citrinin on CYA, with a recorded mean value of 15.6 µg/mL. No detectable citrinin was observed in other examined strains under CYA or YES conditions. Overall, YES medium promoted higher citrinin production than CYA, particularly in *P. citrinum*, highlighting the influence of culture medium composition on mycotoxin biosynthesis.

## 4. Discussion

The present study provides one of the most detailed assessments to date of fungal diversity and multi-mycotoxin contamination in both fresh and dried chili peppers produced and marketed in Guangzhou, southern China. It should be emphasized that the fungal isolation, in vitro mycotoxin production assays, and quantification of mycotoxins in chili samples were conducted as independent studies. Thus, no causative linkage between strain and sample was assumed, nor was a molecular or quantitative relation between certain fungal isolates and the content of one or several mycotoxins in the corresponding chili samples demonstrated. Consequently, the inferences drawn are associations, not causality. The results demonstrate that chili peppers function as highly conducive substrates for a wide array of toxigenic fungi, particularly species of *Fusarium* and *Penicillium*. While *F. concentricum*, *F. fujikuroi*, *F. sulawesiense*, *F. commune*, and *F. verticillioides* have been previously documented on chili peppers [7,102,103,104], this study reports seven species for the first time globally on this host: *F. annulatum*, *F. compactum*, *F. pernambucanum*, *F. ramsdenii*, *F. tardichlamydosporum*, *P. citrinum*, and *P. steckii*. This expanded diversity, including *F. annulatum*, *F. commune*, *F. compactum*, *F. concententricum*, *F. fujikuroi*, *F. pernambucanum*, *F. ramsdenii*, *F. sulawesiense*, *F. tardichlamydosporum*, *F. verticillioides*, *P. citrinum*, and *P. steckii*, underscores the high diversity of fungal communities colonizing chili peppers in this region. These observations align with global evidence that *Capsicum* fruits, like many horticultural commodities, are predisposed to colonization by multiple pathogenic and spoilage fungi along the farm-to-market continuum [23,105,106]. We selected 23 strains for targeted mycotoxin production tests based on their taxonomic relevance (species with known FB_1_ or CIT potential) and on their frequency of isolation (the most common from symptomatic samples). This targeted approach introduces selection bias that may overestimate the prevalence of toxigenic strains, but it efficiently assesses the toxigenic capacity of the species most relevant to observed contamination patterns. In addition, toxigenic potential varied across species and culture conditions, with certain *Fusarium* isolates capable of producing FB_1_ on PDA. In contrast, *Penicillium* species, especially *P. citrinum* and *P. steckii*, exhibited clear citrinin production on both CYA and YES media, indicating that the type of substrate plays an important role in regulating mycotoxin biosynthesis and, consequently, potential food safety risks. It is important to emphasize that while in vitro toxicity, particularly FB_1_ production by *Fusarium* species and CIT by *Penicillium* species on PDA, YES, and CYA aligns with mycotoxin profiles in chili samples, this establishes biological plausibility, not causality. Linking specific isolates to contamination would require targeted molecular approaches (e.g., qPCR of biosynthetic genes), which we acknowledge as a future research direction.

A major finding from this study is the high frequency of multi-mycotoxin contamination, in which DON, AFB_1_, OTA, FB_1_, ZEN, and citrinin were detected simultaneously in both fresh and dried peppers. Such co-contamination implies a diverse fungal microbiome and no single-species attack. Also, in sweet peppers, paprika, and other spices, the occurrence of overlapping toxins has been reported in earlier work, produced simultaneously by toxigenic *Fusarium* and *Penicillium* species, and both toxins could be found together in a food [106,107]. This emphasizes the need for analysis using multi-toxin screening systems, since single-mycotoxin risk assessment underestimates the combined and synergistic risks inherent in mixed toxicity [35]. Toxic interactions among mycotoxins are not well understood at low chronic doses present in the daily diet [108].

The dominance of *Fusarium* species in fresh chili pepper samples corresponds with their ecology as highly aggressive plant pathogens that can invade crop tissues at different stages of crop development. *Fusarium* infections on *Capsicum* plants are reported to cause severe symptoms and wilting, leaf yellowing, and fruit rot with vascular discoloration, resulting in a serious reduction of yield as well as marketable fruit [109,110]. Especially worrisome are species such as *F. verticillioides* and *F. fujikuroi*, which are known to produce fumonisins and other mycotoxins associated with cancer, immunotoxicity, and gastrointestinal toxicity [111,112,113]. The universal detection of DON in all isolates from both fresh and dried peppers further emphasizes the metabolic flexibility of trichothecene-producing *Fusarium* species, which are known to synthesize these toxins early during host colonization [114]. Earlier research in China revealed significant *Fusarium* contamination in sweet peppers [7], supporting the trends observed here, given the agronomic and ecological similarities between sweet and chili pepper cultivars. A unique challenge posed by *Fusarium* species is their ability to produce multiple mycotoxins simultaneously, often within the same substrate. This study contributes to the growing evidence that *Fusarium* can co-produce an array of compounds—including fumonisins, trichothecenes, moniliformin, beauvericin, zearalenone, and [115,116]—leading to complex contamination patterns that complicate detection and risk management. Even at trace levels, these toxins can exert significant toxic effects, and their concurrent presence is increasingly recognized as a major limitation to traditional mycotoxin control strategies [117,118].

*Penicillium* species, in contrast, were more abundant in dried chili peppers—consistent with their ability to thrive under low-water-activity storage conditions. *P. citrinum*, the dominant species identified, is a well-established producer of citrinin, a nephrotoxin implicated in renal impairment and oxidative damage [119,120]. Improper drying of chili peppers creates microenvironments with elevated humidity, facilitating *Penicillium* colonization and citrinin production [121]. This pattern is consistent with surveys in India, Korea, and Europe, where Penicillium was consistently identified as a major colonizer of stored peppers and paprika products [106,122,123]. The higher contamination levels in dried peppers reinforce the importance of controlled drying and storage, especially in regions where sun-drying remains a primary postharvest method.

Multiple recent studies consistently indicate that multi-mycotoxin contamination and co-occurrence are widespread across food and feed commodities, and advances in analytical technologies have considerably improved the detection of these complex toxin mixtures. Mohammedi-Ameur et al. [124] reported the simultaneous presence of major mycotoxins—including aflatoxins (AFs), OTA, DON, and fumonisins (FBs)—alongside emerging mycotoxins in large-scale commodity surveys, indicating that single-toxin surveillance substantially underestimates real exposure. Similarly, Annunziata et al. [125] observed that a high proportion of feed samples contained two or more regulated mycotoxins, with frequent co-occurrence of DON + FBs and AFB_1_ + FB_1_ in the same samples. Expanding this global perspective, Gruber-Dorninger et al. [126] documented that more than 90% of aquaculture feeds and plant-based feed ingredients were contaminated with at least one mycotoxin and that co-contamination involving multiple toxin classes was highly prevalent. Region-specific cereal surveys by Penagos-Tabares et al. [127] highlighted concerning non-compliance rates for aflatoxins and fumonisins in maize harvests from 2021 to 2024, with pronounced effects of climate variability and contamination patterns across harvest years. In support of these findings, pan-regional analyses by Freitag et al. [128] showed that annual weather fluctuations considerably influence multi-mycotoxin occurrence profiles, with drought years showing particularly high co-occurrence of aflatoxins and fumonisins, thereby complicating risk prediction models. Concurrently, method development studies by El-Khatib et al. [41] and Giannioti et al. [129] have expanded LC–MS/MS multi-analyte platforms to detect dozens of regulated and emerging mycotoxins, demonstrating that complex mixtures of regulated, masked, and emerging toxins are routinely present in single samples. The toxicological relevance of these mixtures has been reinforced by experimental studies, with Ochieng et al. [130] reporting that combined exposure to AFB_1_ and FB_1_ produces more severe adverse health effects in animal models than exposure to toxins alone and that mitigation strategies targeting only one toxin often leave other contaminants unaddressed. Furthermore, regional monitoring and exposure assessment studies by Sabillón et al. [131] and Nešić et al. [40] have quantified human and animal co-exposures and emphasized the analytical and regulatory challenges posed by masked and emerging mycotoxins.

Comparable contamination patterns have been widely reported in dried fruits, which frequently harbor multiple mycotoxins at significant concentrations. In Turkish dried figs, Heperkan et al. [132] identified the co-occurrence of OTA (0.1–15.3 ng/g), fumonisin B_1_ (FB_1_) (0.05–3.65 µg/g), and aflatoxins (0.1–763.2 ng/g) within the same samples. A subsequent LC–MS/MS investigation by Sulyok et al. [133] revealed extremely high levels of OTA (up to 11,400 µg/kg), tenuazonic acid (TeA) (18–299,000 µg/kg), and FB_1_ (13–1430 µg/kg) in individual Turkish fig samples. In China, Wei et al. [134] analyzed 220 dried fruit samples—including raisins, dried apricots, dates, and wolfberries—and reported frequent occurrence of *Alternaria* toxins such as TeA, tentoxin (TEN), and mycophenolic acid (MPA), with 31.4% of samples containing two to four toxins simultaneously. In Egyptian dried dates, Abdallah et al. [135] detected OTA levels as high as 6070 µg/kg alongside FB_2_ (16.2 µg/kg) and AFB_1_ (14.4 µg/kg) in the same samples. Similarly, Galván-Romero et al. (2022) [136] observed the co-detection of AFB_1_ (up to 75 µg/kg) and OTA (up to 50 µg/kg) at different stages of the dried fig value chain in Spain. A comprehensive review by Gilbert and Senyuva [137] further confirmed that dried figs frequently contain multiple mycotoxins simultaneously, including AFB_1_, AFB_2_, AFG_1_, AFG_2_, OTA, fumonisin B_2_, and kojic acid, with contamination levels varying widely depending on geographical origin and processing methods.

DON was detected in both fresh and dried peppers; however, higher levels were found in dried samples, possibly reflecting its stability and the early onset of its biosynthesis during initial tissue invasion. The robustness of DON under varying environmental conditions has been reported previously and is linked to the physiology of trichothecene-producing *Fusarium* species [111,114]. The detection of fumonisin B_1_ and zearalenone in fresh samples suggests that toxin accumulation may begin in the field and continue postharvest if environmental conditions remain favorable. Similar observations were made in sweet peppers from China, where preharvest *Fusarium* infections were identified as the main source of mycotoxin contamination [7].

Citrinin production by *Penicillium* in this study was considerably influenced by the culture medium, with both *P. citrinum* and *P. steckii* producing higher levels of citrinin on YES than on CYA, suggesting that nutrient composition plays a critical role in regulating secondary metabolite biosynthesis. These findings are consistent with earlier reports indicating that citrinin production varies widely among *Penicillium* species and depends on growth conditions, with Bragulat et al. [138] reporting citrinin production in *P. expansum*, *P. citrinum*, and *P. verrucosum* on both CYA and YES. In contrast, no production was detected in numerous *Aspergillus* species. The strong toxigenic capacity of *P. citrinum* has also been highlighted through rapid screening methods, including coconut cream agar, which confirmed its reliability as a citrinin producer [139]. The higher citrinin yields observed on YES in the present study may be largely attributed to the high sucrose content of this medium, which provides an easily metabolizable carbon source that enhances fungal growth and stimulates the polyketide biosynthetic pathway responsible for citrinin formation. This observation is further supported by Davis et al. [140], who documented maximum citrinin production on sucrose- and yeast extract–rich media. In contrast, comparatively lower production has been reported on potato-based media for several fungi [141]. Overall, these findings indicate that carbon source availability, particularly sucrose, influences citrinin biosynthesis and may contribute to the risk of toxin accumulation under nutrient-rich conditions.

The results of the present study showed that PDA supported only low-level FB_1_ production by the tested *Fusarium* species, with *Fusarium verticillioides* being the most potent producer. In contrast, *F. annulatum* showed much lower levels, and other species produced only trace or non-detectable amounts, suggesting that PDA is not a highly favorable substrate for strong FB_1_ biosynthesis. This observation is consistent with previous findings that culture medium composition considerably influences mycotoxin production, as shown for citrinin, where Mohamed et al. [139] reported efficient detection on Coconut Cream Agar after 4–5 days of incubation, demonstrating the importance of substrate specificity for toxin expression, while Davis et al. [140] recorded very high citrinin yields (up to 1.75 g/L) on a sucrose–yeast extract medium, indicating that rich carbon–nitrogen sources greatly enhance toxin synthesis. In contrast, Gu et al. [141] reported that potato dextrose medium yielded the lowest citrinin production among several tested media for *Monascus anka*. However, measurable toxin levels were still detected, which closely parallels the present findings, where PDA supported only limited FB_1_ production by *Fusarium* spp. Thereby, these findings suggest that PDA is suitable for screening and comparative evaluation of toxigenic potential. Still, it does not promote high mycotoxin yields, and substrate optimization is essential for maximizing toxin production in laboratory studies.

Environmental conditions appear to be important factors associated with fungal colonization and mycotoxin biosynthesis in chili peppers, with high ambient humidity, elevated temperatures, and inconsistent drying practices likely enhancing fungal proliferation and toxin accumulation [110,142]. Traditional sun-drying, which is widely practiced in rural production systems, may inadvertently increase mycotoxin risks when peppers are exposed for prolonged periods under fluctuating moisture conditions [106,143]. Our findings show that dried peppers contained higher levels of AFB_1_, OTA, FB_1_, ZEN, and citrinin than fresh peppers. Aflatoxin B_1_ and ochratoxin A, which are primarily produced by species of *Aspergillus* and *Penicillium*, are known to be synthesized optimally under fluctuating humidity conditions during drying and storage, providing a plausible explanation for their greater occurrence in dried samples [16,144]. In addition to fungal presence, factors such as the drying environment, plant physiology, and substrate composition may influence mycotoxin biosynthesis [19,145,146], and high humidity during drying can further facilitate both fungal growth and secondary metabolite production. These observations highlight the importance of controlled dehydration systems, in which temperature and relative humidity can be tightly regulated to inhibit microbial proliferation, and reinforce the concept that each stage of processing—fresh handling, drying, and storage—imposes distinct but interconnected risk factors that must be addressed through stage-specific mitigation strategies [147].

Chili peppers represent a clear example of how agricultural production, fungal ecology, and food-safety risks converge, as indicated by the present study, which revealed a complex fungal community dominated by toxigenic *Fusarium* and *Penicillium* species in both fresh and dried peppers marketed in Guangzhou, China. The widespread detection of multiple mycotoxins indicates that chili peppers are highly susceptible to contamination throughout the production and marketing chain. The frequent co-occurrence of multiple mycotoxins, in some cases exceeding international safety limits, indicates potential public health concerns and suggests additive or synergistic toxic effects. These findings emphasize the critical role of postharvest handling, particularly efficient drying and proper storage, in lowering fungal activity and suppressing toxin accumulation. Moreover, the detection of emerging and previously underreported *Fusarium* species suggests that shifting environmental and market conditions may be reshaping fungal populations on chili peppers, reinforcing the need for continuous surveillance and integrated management approaches. Although this study focused on *Fusarium* and *Penicillium*. *Aspergillus* species the main producers of AFB_1_ and OTA, were likely present in the samples but were not successfully identified due to methodological limitations or unfavorable isolation conditions. They were recorded collectively under other genera’ (11% of isolates). Numerous studies have documented toxigenic *Aspergillus* in chili peppers worldwide [8,10,42,148], and their presence during drying and storage explains the detection of these toxins.

This section compares the mycotoxin concentrations and prevalence observed in fresh and dried chili peppers from the present study with data reported in other relevant literature (Table 14). A prominent finding in our study was that dried chili samples had 2- to 10-fold higher levels of toxins than fresh ones. Our results offer a broader spectrum of contamination than previously described: DON, AFB_1_, OTA, and FB_1_ were detected at 100% in dried samples; however, previous reports have reported different patterns of contamination.

Venkatachalapathi et al. [148], who conducted the first preharvest study in India found 48% of fresh red chili samples contaminated with AFs (AFB_1_: 0.4–20 µg/kg) and observed differences among various agro-ecological zones. Su et al. [149] observed a 100% AFs contamination rate in dried red chili pepper, but the levels were many times lower (≤7.19 µg/kg). San Phyo et al. [42] have also reported AFs (97%) and OTA (91%) in dried chili from Myanmar, with values ranging from 0.25 to 72.8 µg/kg for AFs and between 1.7−139 µg/kg for OTA as levels of contamination; out of them, the percentage (56.1%) exceeds the EU limits on maximum levels for aflatoxins. Akintola et al. [150] detected AFB_1_ in 100% of dried chili products (pods, flakes and powder) from Oman at levels up to 19.4 ppb with 34% of powder samples above the threshold value of 10 ppb. Chen et al. [152] found OTA in dried red chilies from China at levels of up to 54.12 µg/kg (peel) and 36.67 µg/kg (seed), with 22.5–34.7% exceeding the EU limit of 20 µg/kg. The amount of AFB_1_ (ND–39. 12 µg/kg), OTA (ND–39. 79 µg/kg), and ZEN (ND–11. 16 µg/kg). In red pepper imported from Turkey, a number of the samples exceeded the limits established by the Turkish Food Codex. Lasram et al. [154] detected AFB_1_ in 90% and AOT in 80% of red chili powder samples from Tunisia (AFB_1_: 0.1 ranging from to 27.1 µg/kg; OTA: ranged from 0.5 to 35.2 µg/kg). Iqbal et al. [155] also reported AFB_1_ (up to 11.7 µg/kg) and OTA (30.4 µg/kg) in red chili sauce from Pakistan, with 44.8% samples exceeding EU limits for AFB_1_ and 23% of the samples surpassing EU levels for OTA. Pickova et al. [9] analyzed paprika samples from the Czech Republic, 35% of which contained OTA, and AFs were detected in 10%, all being below EU maximum limits.

Importantly, the mean estimated DON concentration found in our study was highest (0.56 ± 0.51 µg/g; maximum 1.74 µg/g), and data on this mycotoxin in chili matrices have rarely been reported and analyzed in previous work, indicating a critical control point during the drying process.

In fresh analyzed samples, the contamination profile was significantly different: CIT predominated with the prevalence of 100% at a low level (0.019 ± 0.009 µg/g), whereas OTA was completely absent. In contrast to the studies by Lasram et al. [154] and San Phyo et al. [42], who observed OTA in 80–91% of dried chili products, indicating that OTA contamination occurs mainly during post-harvest processing and drying rather than at the field level.

Dry matrices were favorable for achieving good reproducibility (lower RSDs), and fresh samples exhibited issues, particularly when considering the strong matrix effect on CIT (ME = 88.3%) and low recovery of AFB_1_ (43.29%), which suggest that analytical methods must be optimized according to matrix contamination for moist samples. Such matrix effects are very seldom reported in the literature, where most studies refer only to dried products (cf. Table 11 and Table 12). This is the first report on a systematic comparison of mycotoxin contamination between fresh and dried chili using validated analytical methods optimized for both matrices.

Levels of aflatoxin B_1_ (AFB_1_) in dried chili samples were as high as 180 µg/kg, which is 36-fold greater than the EU maximum tolerance level for *Capsicum* spices (5 µg/kg). The concentrations of ochratoxin A (OTA) also reached 54 µg/kg, which was about 2.7-fold greater than the EU limit (20 µg/kg). These results depict an appreciable level of exceedance of regulatory limits for targeted mycotoxins in dried chili pepper [42,156]. Such high levels of AFB_1_ and OTA may pose public health concerns and underscore the pressing need for enhanced post-harvest management techniques, storage conditions, and ongoing surveillance programs to meet international food safety guidelines.

Ultimately, our findings highlight the importance of stringent control over post-harvest processes to mitigate mycotoxin contamination in the final product. It is noteworthy that this approach entails several methodological limitations. The first limitation of the proposed approach is that fungal isolation, in vitro toxin production, and mycotoxin quantification were performed independently; hence, no direct strain-to-sample linkage was established. Some *Fusarium* and *Penicillium* isolates exhibited toxigenic potential in culture media; however, this observation does not prove that they were responsible for contamination of the original samples. Future work is crucial to adopt qPCR or ddPCR targeting biosynthetic genes (e.g., fum1 and pksCT) to correlate fungal biomass with toxin concentrations in the sample. The second limitation is that isotopically labeled internal standards were not utilized in LC-MS/MS quantification due to budget constraints. Stable isotope dilution assays remain the widespread gold standard for offsetting matrix effects, even though matrix-matched calibration has been fully validated in accordance with SANTE/EC and AOAC guidelines. This constraint was evident in fresh original samples (as received), where higher matrix effects (88.3% for CIT and 43.29% for AFB_1_) were observed. As such, future work also needs to include isotope-labeled internal standards for enhanced accuracy in complex matrices. The third limitation stems from a lack of monitoring of environmental parameters (e.g., temperature, relative humidity, and water activity) during drying and storage. These climatic factors influence fungal proliferation and toxin biosynthesis, and their lack of monitoring limits the predictive power of modeling. Real-time environmental and climate monitoring aspects must be integrated into prospective studies. We anticipate that addressing these limitations will reinforce risk assessment and post-harvest mitigation strategies.

## 5. Conclusions

This study describes the first systematic molecular, morphological, and chemical investigation of fungal contamination and multi-mycotoxin levels in fresh and dried chili peppers marketed in Guangzhou, China. Multi-locus phylogenetic analysis identified a diverse assemblage of *Fusarium* and *Penicillium* species, including several highly toxic taxa not hitherto reported from *Capsicum annuum*, thereby indicating the complexity of the fungal community associated with this crop. LC–MS/MS analysis revealed extensive multi-mycotoxin contamination, with DON detected in all samples and higher levels of AFB_1_, FB_1_, OTA, ZEN, and citrinin in dried peppers, suggesting that the drying process affected toxin concentrations. The frequent occurrence of different mycotoxins, AFB_1_ and FB_1_, at levels well above internationally accepted regulatory limits raises considerable food safety concerns for consumers and chili chains. This research provides an important database for risk-oriented management measures by documenting the toxigenic potency of fungal species isolated from contaminated samples. The results demonstrate the need to improve postharvest drying and storage practices and introduce routine multi-mycotoxin surveys in markets. The presence of newly described and under-researched *Fusarium* species also emphasizes the importance of continued surveillance in response to shifting climates and environments. Lastly, this research contributes to understanding chili pepper fungal ecology and mycotoxin risk, providing a scientific basis for developing improved control strategies, breeding resistant varieties, and strengthening regulatory systems to protect human health and ensure sustainable chili pepper production.

## Figures and Tables

**Table 3:** GenBank accession number of *Fusarium s*pecies sequences used in this study.

Strain	Species				GenBank Accession Number	
		*tub2*	*cmdA*	*tef1-α*	*rpb1*	*rpb2*
ZHKUCC 25-1346	*Fusarium verticillioides*	PX805865	PX805877	PX832374	PX911392	PX842522
ZHKUCC 25-1347	*Fusarium verticillioides*	PX805866	-	PX832375	PX832386	PX842523
ZHKUCC 25-1348	*Fusarium verticillioides*	PX805867	PX805878	PX832376	PX832387	PX842524
ZHKUCC 25-1349	*Fusarium verticillioides*	PX805868	PX805879	PX832377	PX832388	PX842525
ZHKUCC 25-1350	*Fusarium verticillioides*	PX805869	PX805880	PX832378	-	PX842526
ZHKUCC 25-1343	*Fusarium fujikuroi*	PX805870	PX805881	PX832379	-	-
ZHKUCC 25-1342	*Fusarium concentricum*	PX805871	PX805882	PX832380	-	-
ZHKUCC 25-1351	*Fusarium annulatum*	PX805872	PX805883	PX832381	-	PX842527
ZHKUCC 25-1352	*Fusarium annulatum*	PX805873	PX805884	PX832382	PX832389	PX842528
ZHKUCC 25-1353	*Fusarium annulatum*	PX805874	PX805885	PX832383	-	PX842529
ZHKUCC 25-1354	*Fusarium annulatum*	PX805875	PX805886	PX832384	-	-
ZHKUCC 25-1355	*Fusarium annulatum*	PX805876	PX805887	PX832385	-	PX842530
ZHKUCC 25-1345	*Fusarium commune*	PX842531	-	PX842532	-	PX842533
ZHKUCC 25-1357	*Fusarium ramsdenii*	PX842534	PX842535	PX844937	-	PX934423
ZHKUCC 25-1344	*Fusarium sulawesiense*	-	-	PX911383	-	-
ZHKUCC 25-1356	*Fusarium pernambucanum*	-	-	PX911384	-	PX911386
ZHKUCC 25-1359	*Fusarium compactum*	-	-	PX911385	-	PX911387
ZHKUCC 25-1358	*Fusarium tardichlamydosporum*	PX911388	PX911389	PX911390	-	PX911391

**Table 4:** GenBank accession number of *Penicillium* species sequences used in this study.

Strain	Species			GenBank Accession Number	
		*tub2*	*cmdA*	ITS	*rpb1*
ZHKUCC 25-1360	*Penicillium citrinum*	PX832365	PX832356	PX794524	PX805860
ZHKUCC 25-1361	*Penicillium citrinum*	PX832366	PX832357	PX794525	-
ZHKUCC 25-1362	*Penicillium citrinum*	PX832367	PX832358	PX794526	PX805861
ZHKUCC 25-1363	*Penicillium citrinum*	PX832368	PX832359	PX794527	-
ZHKUCC 25-1364	*Penicillium citrinum*	PX832369	PX832360	-	PX805862
ZHKUCC 25-1365	*Penicillium citrinum*	PX832370	PX832361	-	PX805863
ZHKUCC 25-1366	*Penicillium citrinum*	PX832371	PX832362	PX794528	PX805864
ZHKUCC 25-1367	*Penicillium citrinum*	PX832372	PX832363	PX794529	-
ZHKUCC 25-1368	*Penicillium steckii*	PX832373	PX832364	PX794530	-

**Table 5:** Percentage of contaminated samples, average, minimum, maximum, IQR, and confidence intervals for concentrations of toxins in dried chili.

Mycotoxin	No. of Samples Analyzed	No. of Positive Samples	%Contaminated Samples	Average ^a^ (µg/g) ± SD	Minimum ^c^ (µg/g) ± SD	Maximum ^b^ (µg/g) ± SD	IQR	Confidence Intervals (µg/g) 95%
DON	33	33	100%	0.56 ± 0.51	0.074 ± 0.0036	1.74 ± 0.054	0.803	0.38–0.74
AFB_1_	33	33	100%	0.082 ± 0.046	0.015 ± 0.0006	0.17 ± 0.017	0.064	0.066–0.099
OTA	33	33	100%	0.049 ± 0.003	0.047 ± 0.0018	0.054 ± 0.0004	0.0046	0.048–0.051
FB_1_	33	33	100%	0.067 ± 0.042	0.041 ± 0.0026	0.192 ± 0.015	0.016	0.053–0.082
ZEN	33	21	63.63%	0.003 ± 0.0035	0.0008 ± 0.0001	0.012 ± 0.000	0.0025	0.0009–0.004
CIT	33	27	81.81%	0.039 ± 0.019	0.021 ± 0.0009	0.059 ± 0.0007	0.029	0.027–0.041

^a^ Average of the concentrations detected for that metabolite; ^b^ Highest concentration detected; ^c^ Lowest concentration detected. SD: standard deviation. DON: deoxynivalenol; AFB_1_: aflatoxin B_1_; OTA: ochratoxin A; FB_1_: fumonisin B_1_; ZEN: zearalenone; CIT: citrinin; IQR: interquartile range.

**Table 6:** Percentage of contaminated samples, average, minimum, maximum, IQR, and confidence intervals concentration of toxins in fresh chili.

Mycotoxin	No. of Samples Analyzed	No. of Positive Samples	%Contaminated Samples	Average ^a^ (µg/g) ± SD	Minimum ^c^ (µg/g) ± SD	Maximum ^b^ (µg/g) ± SD	IQR	Confidence Intervals (µg/g) 95%
DON	33	21	63.63%	0.087 ± 0.197	0.0064 ± 0.0005	0.68 ± 0.020	0.064	0.017–0.157
AFB_1_	33	3	9.09%	<LOQ	<LOQ	<LOQ	--	--
OTA	33	0	0	--	--	--	--	--
FB_1_	33	24	72.72%	0.004 ± 0.004	0.00156 ± 0.0003	0.013 ± 0.0005	0.007	0.002–0.005
ZEN	33	9	27.27%	0.0006 ± 0.001	0.0002 ± 0.00002	0.0044 ± 0.00007	0.0002	0.0001–0.001
CIT	33	33	100%	0.019 ± 0.009	0.0085 ± 0.00047	0.035 ± 0.0019	0.013	0.016–0.022

^a^ Average of the concentrations detected for that metabolite; ^b^ Highest concentration detected; ^c^ Lowest concentration detected. SD: standard deviation. DON: deoxynivalenol; AFB_1_: aflatoxin B_1_; OTA: ochratoxin A; FB_1_: fumonisin B_1_; ZEN: zearalenone; CIT: citrinin; IQR: interquartile range; <LOQ: detected but below the limit of quantification.

**Table 7:** Analytical precision of mycotoxin measurement in dried chili pepper (dried samples).

Treatment	DON		AFB_1_		OTA		FB_1_		ZEN		CIT	
	STD	RSD	STD	RSD	STD	RSD	STD	RSD	STD	RSD	STD	RSD
A	0.004	3.671	0.003	8.999	0.002	4.47	0	0.692	0	0	0.002	4.09
B	0.006	5.441	0.001	3.782	0.001	2.22	0.004	8.391	0	0	0.001	4.61
C	0.007	3.478	0.005	5.955	0.003	4.9	0.004	7.75	0.0002	9.95	0.001	1.24
D	0.066	6.719	0.012	12.217	0.002	3.94	0.007	10.634	0.0001	1.44	0	0
E	0.034	7.172	0.01	7.773	0.002	3.63	0.008	13.117	0	0	0	0.24
F	0.016	2.113	0.002	3.519	0	1.03	0.015	7.949	0	0	0.002	3.55
G	0.068	6.631	0.006	8.222	0.005	9.67	0.003	6.432	0.0001	12.03	0.002	5.08
H	0.054	3.119	0.018	18.025	0	0.74	0.007	10.723	0.0001	3.54	0.003	7.74
I	0.001	0.842	0.017	9.684	0.001	2.32	0.002	3	0	4.06	0.001	1.95
J	0.004	4.936	0.007	18.569	0.001	1.24	0.009	16.949	0	0	0	0
K	0.014	2.627	0.015	16.425	0.001	2.37	0.003	5.068	0.0001	8.18	0.003	8.5

DON: deoxynivalenol; AFB_1_: aflatoxin B_1_; OTA: ochratoxin A; FB_1_: fumonisin B_1_; ZEN: zearalenone; CIT: citrinin; STD: standard deviation; RSD: relative standard deviation. Analytical precision of mycotoxin measurement in dried chili pepper samples across 11 treatments (A–K). Treatments correspond to market sources.

**Table 8:** Analytical precision of mycotoxin measurement in fresh chili pepper (fresh samples).

Treatment	DON		AFB_1_		OTA		FB_1_		ZEN		CIT	
	STD	RSD	STD	RSD	STD	RSD	STD	RSD	STD	RSD	STD	RSD
A	0.004	9.52	0	0	0	0	0.0003	17.98	0	0	0.0039	15.23
B	0.001	1.48	0	0	0	0	0.0004	14.39	0.0001	1.56	0.0003	1.1
C	0.0005	1.78	0	0	0	0	0.0002	7.59	0.00002	9.39	0.0017	5.8
D	0.001	1.09	0	0	0	0	0.0003	5.7	0.0001	5.3	0.00004	0.17
E	0.0203	2.96	0.00004	8.26	0	0	0	0	0	0	0.0016	17.44
F	0	0	0	0	0	0	0.0002	2.23	0	0	0.0019	5.4
G	0.0006	8.7	0	0	0	0	0.0001	1.28	0	0	0.0005	5.52
H	0	0	0	0	0	0	0.0005	3.72	0	0	0.0001	0.97
I	0	0	0	0	0	0	0.0002	14	0	0	0.0012	6.7
J	0.0001	0.16	0	0	0	0	0	0	0	0	0.0014	9.51
K	0	0	0	0	0	0	0	0	0	0	0.0002	2.04

DON: deoxynivalenol; AFB_1_: aflatoxin B_1_; OTA: ochratoxin A; FB_1_: fumonisin B_1_; ZEN: zearalenone; CIT: citrinin; STD: standard deviation; RSD: relative standard deviation. Analytical precision of mycotoxin measurement in dried chili pepper samples across 11 treatments (A–K). Treatments correspond to market sources.

**Table 9:** Analysis of variance of the effects of different treatments (A–K) on the contents of deoxynivalenol (DON), aflatoxin B_1_ (AFB_1_), ochratoxin A (OTA), fumonisin B_1_ (FB_1_), zearalenone (ZEN), and citrinin (CIT) in dried chili (dry weight basis). Treatments correspond to market sources.

S.V	DF	Mean Square (M.S)					
		DON	AFB_1_	OTA	FB_1_	ZEN	CIT
Treatment	10	0.831 **	0.0064 **	0.000019 **	0.0053 **	0.000038 **	0.0012 **
Error	22	0.0013	0.0002	0.000004	0.00015	0.00000001	0.0000026
CV (%)		6.39	8.61	4.07	14.29	3.63	4.7

** Significant at the 1% probability levels.

**Table 10:** Analysis of variance of the effects of different treatments (A–K) on the contents of deoxynivalenol (DON), aflatoxin B_1_ (AFB_1_), ochratoxin A (OTA), fumonisin B_1_ (FB_1_), zearalenone (ZEN) and citrinin (CIT) in fresh chili (fresh weight basis). Treatments correspond to market sources.

S.V	DF	Mean Square (M.S)					
		DON	AFB_1_	OTA	FB_1_	ZEN	CIT
Treatment	10	0.121 **	ND	0	0.00005 **	0.000006 **	0.00025 **
Error	22	0.000039	ND	0	0.00000006	~0	0.0000025
CV (%)		7.21	--	--	6.36	6.83	8.42

** Significant at the 1% probability levels.; ND = not determined due to low detection frequency (<10% of samples); ~0 = variance less than 0.000001; -- = not calculated.

**Table 11:** Validation parameters (Recovery, LOD, LOQ, ME, and R2) were calculated for dried chili.

Mycotoxin	Recovery (%) ± SD	LOD (µg/g)	LOQ (µg/g)	ME (%)	R^2^
DON	100.26 ± 0.82	0.0022	0.0144	18.2	0.9994
AFB_1_	100 ± 0.00	0.0011	0.0074	15.6	0.9997
OTA	100 ± 0.00	0.0004	0.0027	20	0.9997
FB_1_	100 ± 0.00	0.0003	0.0023	28.4	0.9996
ZEN	100 ± 0.00	0.0002	0.0009	0.94	0.9997
CIT	97.88 ± 3.69	0.0007	0.0055	13.9	0.9996

LOD: limit of detection; LOQ: limit of quantification; ME: matrix effect.

**Table 12:** Validation parameters (Recovery, LOD, LOQ, ME, and R2) were calculated for fresh chili.

Mycotoxin	Recovery (%) ± SD	LOD (µg/g)	LOQ (µg/g)	ME (%)	R^2^
DON	75.48	0.0027	0.009	84.4	0.9997
AFB_1_	43.29	0.0013	0.0045	0.5	0.9998
OTA	-	-	-	-	-
FB_1_	75.59	0.0003	0.0009	39.4	0.9997
ZEN	79.04	0.0001	0.0003	6.46	0.9997
CIT	75.09	0.0039	0.0129	88.3	0.9997

LOD: limit of detection; LOQ: limit of quantification; ME: matrix effect.

**Table 13:** Mycotoxin (µg/g) production by *Penicillium* and *Fusarium* species on YES, CYA, and PDA media (n.e. = not evaluated).

Culture Media	YES	CYA	PDA
**Mycotoxins (µg/g)**	**CIT**	**CIT**	**FB_1_**
*P. citrinum*			
No. of positive/total	8/8	7/8	n.e.
MIN	5.16	0	
MAX	303	251.1	
Mean Value	220.08	122.81	
*P. steckii*	108.40	15.6	n.e.
*F. verticillioides*	n.e.	n.e.	
No. of positive/total			4/5
MIN			0
MAX			4.62
Mean Value			1.924
*F. annulatum*	n.e.	n.e.	
No. of positive/total			4/5
MIN			0
MAX			0.34
Mean Value			0.104
*F. fujikuroi*	n.e.	n.e.	0.13
*F. concentricum*	n.e.	n.e.	0
*F. ramsdenii*	n.e.	n.e.	0.16
*F. compactum*	n.e.	n.e.	0.06

**Table 14:** Summary of mycotoxin occurrence in chili peppers and related spices: A cross-study comparison (2021–2026).

Study (Location, Year)	Sample Type	Key Mycotoxins Detected (Incidence)	Concentration Range	Co-Occurrence and Notable Findings
Present Study (China, 2026)	Fresh and Dried Chilies	DON (100%), FB_1_, CIT, AFB_1_, OTA, ZEN	DON: 0.04–1.74 µg/g; AFB_1_: up to 0.18 µg/g	First report of seven fungal species on chili. Drying is associated with 2–10x higher toxin levels.
[148]	Pre-harvest Red Chilies (*Capsicum annuum* L.)	Aflatoxin B_1_ (38.7%), AFB_2_, AFG_1_, AFG_2_ (48% total AF incidence)	AFB_1_: 0.1–20.7 µg/kg; Total AFs: up to 30.5 µg/kg	First comprehensive pre-harvest study in India. *Aspergillus* incidence: 100%. Significant variation across AEZs. Positive correlation between *Aspergillus* abundance and AF levels (ρ = 0.24, *p* < 0.05). Zone-specific environmental drivers > agronomic factors.
[149]	Dried Red Chili Pepper	AFB_1_, AFG_1_ AFG_2_, AFB_2_	AFs: ≤7.19 µg/kg; AFB_1_: up to 4.8 µg/kg	100% AFs positive; 29.2% of *A. flavus* strains are highly toxigenic.
[42]	Dried chili	AFs (97%), OTA (91%)	AFs: 1.8–72.8 µg/kg; OTA: 1.1–139.4 µg/kg	56.1% exceeded EU AFs limit; MOE < 10,000; first OTA report in Myanmar chili.
[150]	Dried Red Chili Pods, Flakes, Powder	AFB_1_ (100%)	Pods: 2.0–9.6 ppb; Flakes: 1.6–19.2 ppb; Powder: 2.2–19.4 ppb	34% of powder samples > 10 ppb; 83.5% of consumers are unaware of aflatoxins
[151]	Chili Powder	AFs, OTA	AFs: <LOD–56.3 µg/kg; OTA: <LOD–24.7 µg/kg	38% co-occurrence of AFs and OTA; estimated daily intake concerning high consumers.
[152]	Dried Red Chilies	OTA	OTA: up to 54.12 µg/kg (peel); up to 36.67 µg/kg (seed)	22.5% of peels and 34.7% of seeds exceeded the EU OTA limit (20 µg/kg).
[153]	Black pepper, red pepper, Cumin, Turmeric	AFB_1_, AFB_2_, AFG_1_, AFG_2_, OTA, ZEN	AFB_1_: ND–39.12 µg/kg; AFB_2_: ND– µg/kg AFG_1_: ND–0.92 µg/kg; AFG_2_: ND–3.67 µg/kg; OTA: ND–39.79 µg/kg; ZEN: ND–11.16 µg/kg	140 samples exceeded TFC limits for AFB_1_ and total aflatoxins: red pepper was the most contaminated
[154]	Red Chili Powder	AFB_1_ (90%), OTA (80%)	AFB_1_: 0.1–27.1 µg/kg; OTA: 0.5–35.2 µg/kg	5.4% exceeded the EU AFB_1_ limit; 3.6% exceeded the EU OTA limit.
[155]	Red Chili Sauce	AFB_1_, Total AFs, OTA	AFB_1_: up to 11.7 µg/kg; Total AFs: up to 26.6 µg/kg; OTA: up to 30.4 µg/kg	44.8% exceeded the EU AFB_1_ limit; 23% exceeded the EU OTA limit.
[9]	Spices (Paprika)	OTA (35%), AFs (10%)	OTA: 0.3–15.3 µg/kg; AFB_1_: <LOD–2.1 µg/kg	OTA was the predominant mycotoxin. All OTA-positive samples were below EU ML.

## Data Availability

The original contributions presented in this study are included in the article. Further inquiries can be directed to the corresponding author(s).
